# Spatiotemporal and Interannual Habitat Variability of Three *Charybdis* Swimming Crab Species (Brachyura: Portunidae) in the Southern Yellow Sea and East China Sea

**DOI:** 10.3390/biology15161406

**Published:** 2026-08-17

**Authors:** Min Xu, Yong Liu, Hongmei Li, Qi Zhao, Lijian Xue, Qiang Wu, Jianzhong Ling, Huiyu Li

**Affiliations:** 1 East China Sea Fisheries Research Institute, Chinese Academy of Fishery Sciences, Shanghai 200090, China; xumin@ecsf.ac.cn (M.X.); liuy@ecsf.ac.cn (Y.L.); 2 Shanghai Society of Oceanology and Limnology, East China Sea Ecology Center, Ministry of Natural Resources, Shanghai 200062, China; limailg@126.com; 3 Fisheries College, Ocean University of China, Qingdao 266003, China; tozhaoqi@163.com; 4 School of Fisheries, Zhejiang Ocean University, Zhoushan 316022, China; h01007@zjou.edu.cn; 5 Yellow Sea Fisheries Research Institute, Chinese Academy of Fishery Sciences, Qingdao 266071, China; wuqiang@ysfri.ac.cn

**Keywords:** Boosted Regression Trees, crustacea, Decapoda, East China Sea region, East Asia, habitat suitability model, portunoidea, Tweedie Generalized Additive Model

## Abstract

*Charybdis (Gonioneptunus) bimaculata* (Miers, 1886), *C. (Charybdis) japonica* (A. Milne-Edwards, 1861), and *C. (Charybdis) miles* (De Haan, 1835) are dominant target species harvested by small-scale artisanal fisheries in the coastal waters of China. Characterizing their spatial distribution and interannual population variability is critical for sustainable crustacean stock conservation. Previous investigations of these three *Charybdis* congeners have been limited to short-duration, single-season, or geographically restricted descriptive surveys, with no systematic cross-species comparison of seasonal habitat use. This has created major knowledge gaps regarding their long-term spatiotemporal dynamics and ecological niche partitioning. We conducted standardized bottom-trawl surveys aboard research vessels across the southern Yellow Sea and East China Sea from 2018 to 2021 to quantify numerical abundance and biomass of the three congeneric crabs. Two species distribution modeling frameworks—Generalized Additive Models (GAMs) and Boosted Regression Trees (BRTs)—were implemented to generate fine-resolution seasonal habitat suitability maps for each species. We identified minimum thermal thresholds for species presence: bottom water temperature > 8 °C for *C. bimaculata* and >10 °C for *C. miles*. Core aggregation zones of *C. bimaculata* and *C. japonica* occurred in waters adjacent to the Yangtze River Estuary (32–34° N), whereas *C. miles* predominated in offshore habitats of the southern East China Sea (27–31° N). In this study, *C. bimaculata* favors shallow seas with warm bottom water and euryhaline conditions, *C. japonica* prefers low-salinity coastal waters with cold bottom water, and *C. miles* thrives in deep, high-salinity offshore waters with eurythermal tolerance. The findings of this study can contribute to the sustainable fisheries management and conservation of these species.

## Simple Summary

*Charybdis (Gonioneptunus) bimaculata* (Miers, 1886), *C. (Charybdis) japonica* (A. Milne-Edwards, 1861), and *C. (Charybdis) miles* (De Haan, 1835) are commercially valuable swimming crabs distributed across China’s marginal seas. The stock status and population abundance of these crabs directly support the livelihoods of coastal fishers and underpin the socioeconomic stability of coastal fishing communities. Quantifying species-specific abundance and distribution patterns across fine spatiotemporal scales is therefore a research priority for crustacean fishery governance. The findings presented in this study provide actionable scientific evidence for the conservation, sustainable management, and spatial planning of coastal swimming crab fisheries.

## 1. Introduction

Crustacean fisheries constitute a vital component of global commercial harvests and represent the fastest expanding fishery sector worldwide [1]. Swimming crabs are captured using an extensive suite of fishing gears—including bottom trawls, otter trawls, stow nets, gillnets, trammel nets, set nets, hoop nets, and baited pots—across habitats ranging from intertidal flats to offshore continental shelf waters [2]. These portunid crabs fulfill dual ecological and socioeconomic functions within marine shelf ecosystems, sustaining the livelihoods of coastal small-scale fishers [3]. As benthic crustaceans with limited swimming capacity, they cannot undertake long-distance migratory movements [4]. Annual evaluations of seasonal spatial distribution patterns for three congeneric *Charybdis* swimming crabs (*C. bimaculata*, *C. japonica*, *C. miles*; Eubrachyura: Portunidae) are indispensable to inform targeted stock conservation and fishery regulatory measures. These three species are selected for targeted evaluation because they constitute ecologically important benthic predators in coastal shelf ecosystems and simultaneously represent key commercial crustacean resources supporting regional small-scale fisheries.

The two-spotted swimming crab, *Charybdis bimaculata*, occurs across China’s marginal seas (including waters around Taiwan Island) and a broad suite of overseas regions: Japan, the Republic of Korea, Australia, India, the Maldives Archipelago, and South Africa [5]. It occupies coastal habitats with muddy, sandy, and mixed mud–sand seafloors abundant in shell hash, occupying depths spanning the intertidal zone down to 439 m [6]. With a lifespan of 18 months, *C. bimaculata* serves as an important food source for demersal fishes, such as *Pleuronichthys cornutus* and *Conger myriaster* [7]. The soldier swimming crab, *Charybdis miles*, is a warm-water, medium-bodied commercially harvested portunid with broad thermal and salinity tolerance (i.e., eurythermal and euryhaline) [8]. Its primary distribution covers the East China Sea (south of the Yangtze River Estuary) and South China Sea, with additional populations recorded in waters off Japan, Australia, the Philippines, Singapore, India, and the Gulf of Oman [9]. *C. miles* is found in mud, mud–sand, sand, and sandy–shell mixed seafloor substrates [9]. This species produces multiple ovigerous broods and undergoes batch spawning throughout a single reproductive cycle [8].

The Japanese paddle crab, *Charybdis japonica*, is a medium-sized commercially valuable swimming crab with warm-temperate biogeographic affinity, distributed widely across coastal waters of China, the Republic of Korea, Japan, Malaysia, and the Red Sea basin [10]. It occupies estuarine environments characterized by muddy, sandy, rocky, and reef substrates, at depths ranging from 9 to 45 m [11]. *C. japonica* is an omnivorous benthic predator whose primary diet comprises crustaceans, diatoms, macroalgae, bivalves, polychaetes, and small fish, supplemented by gastropods and echinoderms [12]. Off southern Fujian, female *C. japonica* reach sexual maturity at a minimum carapace width of 48 mm and a minimum wet body mass of 24 g [10].

Species distribution modelling (SDM) has emerged as a core analytical framework for quantitative stock assessment in marine ecosystems globally [13]. Traditional fishery-independent trawl surveys only generate discrete, snapshot abundance records at spatially limited sampling stations, making it impossible to generate continuous fine-resolution habitat maps for target species across broad continental shelf domains. SDM frameworks integrate long-term standardized trawl survey datasets and multi-dimensional environmental covariates to interpolate species occurrence and abundance over un-sampled areas, explicitly revealing seasonal and interannual spatial heterogeneity of fishery populations [14]. Generalized Additive Models (GAMs) are among the most prevalent statistical tools in fishery ecology, as they uniquely resolve complex non-linear relationships between aquatic organism abundance and continuous environmental gradients—relationships that standard linear regression cannot reliably capture [15]. No comparable published research has systematically analyzed and compared the ecological niches of the three *Charybdis* crab species within our study area.

On the continental shelf of the East China Sea, *C. bimaculata* makes up 15% of the total crab landings. Together with *C. japonica*, these two species dominate the local crab assemblage [16]. *C. miles* ranks among the five most commercially valuable crab species in the Mindongbei Fishing Ground, where its landings make up more than 90% of the total crab harvest from this fishery [9]. Although the three congeneric *Charybdis* crabs have been consistently recorded across these shelf waters, comprehensive systematic biological and ecological datasets remain scarce. Single-species targeted surveys have failed to fully characterize the spatial population dynamics of entire crab assemblages. Unlike previous descriptive surveys restricted to short-duration sampling, single-season coverage, and geographically limited study regions [5,8,10], we conducted standardized grid-based bottom-trawl surveys across the continental shelf over three consecutive full annual cycles, encompassing all four seasons. The fine-resolution seasonal habitat suitability maps produced in this study mitigate critical drawbacks of existing uniform, one-size-fits-all marine fishery regulatory policies. Our modeling outputs support the development of regionally and seasonally differentiated fishing moratorium frameworks, which are customized to match species-specific spawning and juvenile nursery hotspots.

This study aimed to quantify seasonal spatial distribution patterns, interannual population fluctuations, and species-specific environmental responses of *C. bimaculata*, *C. japonica*, and *C. miles* in the southern Yellow Sea and East China Sea over the 2018–2021 survey period, with a focus on depth, bottom temperature, and salinity gradients. We employed Generalized Additive Models (GAMs) and Boosted Regression Trees (BRTs) to map fine-scale seasonal habitats of the three crabs. We selected the dataset spanning 2018–2021 because new fishing gear was fully implemented starting in November 2018, while survey stations in 2022 were sparsely distributed due to restrictions associated with the COVID-19 pandemic. This multi-species, multi-year continuous comprehensive dataset fills the research gap regarding the seasonal spatiotemporal distribution patterns of the three commercially valuable crab species. The findings presented in this study deliver implementable, spatially refined scientific evidence for the sustainable management of crab fisheries.

## 2. Materials and Methods

### 2.1. Study Area and Data Collection

Fishery-independent bottom-trawl data for the three *Charybdis* species were collected across eight sequential research cruises covering the southern Yellow Sea and East China Sea between 2018 and 2021. The surveys were carried out in spring (22 April–10 May 2019, 141 stations; 7 May–17 June 2020, 143 stations; 22 April–19 June 2021, 87 stations), summer (13 August–27 September 2019 and 2020, both 140 stations), autumn (2–11 November 2018, 127 sampling stations; 2 November 2021–4 January 2022, 105 stations), and winter (4–27 January 2019, 111 stations). Field samplings were performed using research vessels Zhongkeyu 211 and Zhongkeyu 212. All tows deployed a standardized trawl net with a 102-mesh opening, 72.24 m headline, 82.44 m ground rope, and 20 mm cod-end mesh. Each station received a single 1 h tow at a consistent towing speed of 3–4 knots. Only one trawl operation was conducted at each sampling station.

**Figure 1:**
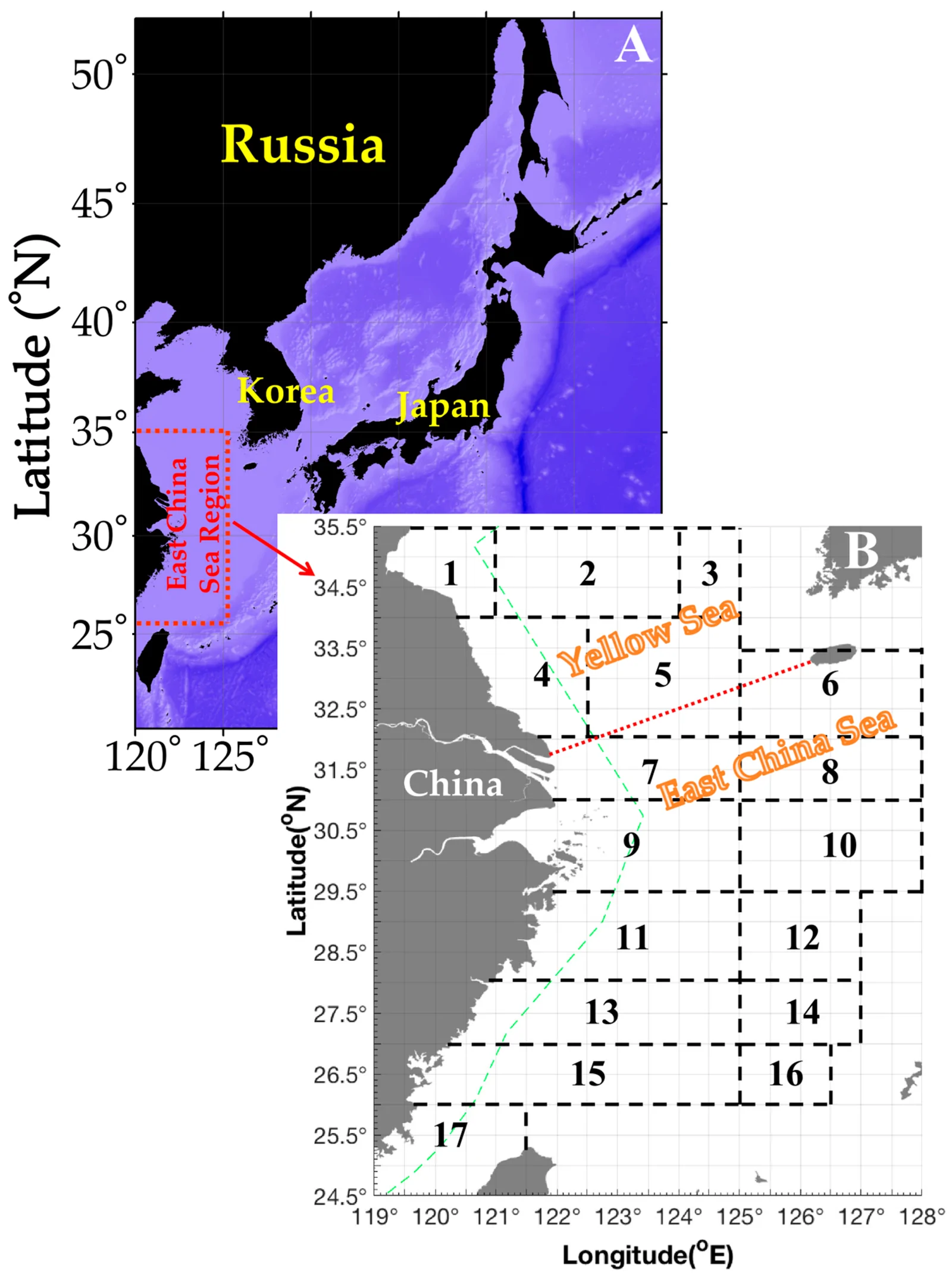
Map of the study domain and stratified trawl survey fishing grounds. Panel (**A**) shows the full geographic extent of the survey area covering the southern Yellow Sea and East China Sea of China (26.50° N–35.00° N, 120.00° E–127.00° E) in East Asia, demarcated by the red dashed rectangular boundary. Panel (**B**) delineates 17 standardized fishing ground strata: (1) Haizhou Bay, (2) Lianqingshi, (3) Liandong, (4) Lvsi, (5) Dasha, (6) Shawai, (7) Yangtze River Estuary, (8) Jiangwai, (9) Zhoushan, (10) Zhouwai, (11) Yushan, (12) Yuwai, (13) Wentai, (14) Wenwai, (15) Mindong, (16) Minwai, (17) Minzhong. The green dashed line marks the motor-trawl prohibition line. The red dashed line indicates the boundary between the Yellow and East China Seas.

The survey area extended from 26°30′ N to 35°00′ N latitude and 120°00′ E to 127°00′ E longitude, with sampling stations arranged in a grid pattern at 30 min intervals of latitude and longitude (Figure 1). A snake-shaped sampling route was adopted across all seasons. Surveys in the region south of 30°00′ N were conducted from north to south, while those north of 30°00′ N were performed from south to north. Species identification was completed in the laboratory to confirm the occurrence of the three crab species at each station. All individuals were counted and weighed to the nearest 0.1 g for wet weight. Two indices were used to quantify catch per unit time: biomass-based CPUE (unit: g·h^−1^) and individual-based CPUE (unit: ind·h^−1^).

Catch per unit effort (CPUE) was computed according to the equations presented below: CPUE_w_ = *W_i_* /*t_i_* and CPUE_n_ = *N_i_* /t_i_, where *N_i_* is the number of individuals caught at station *i* (ind), *W_i_* is the catch weight at station *i* (g), and *t_i_* is the trawling duration at station *i* (h). Furthermore, the average individual weight (AIW) at each station was defined as the ratio of CPUE by weight (CPUE_w_) to CPUE by number (CPUE_n_). In addition, we measured the carapace length (unit: mm) of *C. bimaculata* specimens sampled in the study area in May 2019, August 2019, September 2019, September 2020, August 2023, September 2023, January 2024, April 2025 and January 2026, and identified whether ovigerous females were present. All Kruskal–Wallis tests and post hoc Dunn–Bonferroni pairwise comparisons were performed on station-level standardized CPUE_n_ data (including zero-catch stations), with each trawl station treated as an independent replicate. Aggregated survey means or positive-catch-only subsets were not used, as zero catches reflect true habitat unsuitability and are an ecologically meaningful component of species distribution patterns.

In situ hydrographic covariates (depth, temperature, salinity) were measured at every station using a SeaBird SBE19 conductivity–temperature–depth (CTD) profiler (SeaBird Scientific, Bellevue, WA, USA). Sea surface temperature (SST) and sea surface salinity (SSS) were sampled within the uppermost 3 m of the water column. Seabed temperature (SBT) and seabed salinity (SBS) were logged 2 m above the seafloor at stations < 50 m depth, and 2–4 m above the seafloor at deeper stations.

### 2.2. Habitat Suitability Modeling Using Tweedie GAMs and Boosted Regression Trees

We selected three species of swimming crabs of the genus *Charybdis* as study species, and collected datasets including spatial coordinates (longitude and latitude), temporal information (different years and seasons), abundance, and environmental factors in the survey area: SST, SSS, SBT, SBS, and depth. Missing environmental data were only found at two sampling stations from the January 2019 survey cruise. We manually excluded these records before executing the analytical scripts, leaving a total of 109 valid station records for the three crab species surveyed in January 2019. Year and season were defined as categorical factors to construct a standardized analytical dataset.

Tweedie Generalized Additive Models (GAMs) were fitted to zero-inflated numerical CPUE data to generate univariate environmental Suitability Indices (SIs) for each hydrographic predictor (SST, SSS, SBT, SBS, depth). The complete model structure was defined as

*g*(*µ*) ~ *s*(*env*_*var*, *k* = 4, *bs* = ‘*cr*’) + *s*(*lon*, *lat*, *bs* = ‘*tp*’) + *season* + *s*(*year*, *bs* = ‘*re*’) (1)

where *µ* denotes expected numerical CPUE at each station; the Tweedie distribution family was selected with a natural log link function; *s*(*env_var*, *k* = 4, *bs* = ‘*cr*’) = one-dimensional penalized cubic regression spline for each environmental covariate, with a basis dimension of *k* = 4 which is set to constrain the smoothing degrees of freedom to prevent overfitting; *s*(*lon*, *lat*, *bs* = ‘*tp*’) = two-dimensional thin-plate spline for spatial smoothing to mitigate spatial autocorrelation across sampling locations; season = categorical fixed effect representing seasonal variation; and *s*(*year*, *bs* = ‘*re*’) = random smooth term for year to account for interannual variability, a modeling choice designed to mitigate partial confounding between unbalanced seasonal sampling years and intrinsic seasonal abundance cycles. The model adopted the Tweedie distribution to accommodate zero-inflated catch data, while separating fixed seasonal signals from year-to-year stochastic deviations.

Based on the model predictions, the min–max normalization method was applied to scale the single-variable response values into the range of 0–1, yielding the Suitability Index (SI) for each environmental factor. An SI value closer to 1 indicates that the corresponding environmental conditions are more favorable for the survival and distribution of the target species. Concurvity (multicollinearity between penalized smooth terms) was quantified using the concurvity() function in the R mgcv package with the argument full = TRUE. Maximum and mean concurvity metrics were extracted for each environmental covariate smooth function. A threshold of 0.8 was implemented to flag severe concurvity, with predictors exceeding this cutoff classified as carrying high multicollinearity risk. The gam.check() function was executed iteratively for each environmental predictor to generate a four-panel diagnostic figure suite, including fitted value vs residual scatter, residual Q-Q normal plots, residual histograms, and basis dimension validity checks. Custom Pearson residual vs fitted value scatter plots were employed to visually identify heteroscedasticity and overdispersion. Horizontal reference lines at ±2 standard deviations were added to highlight extreme residuals. The sufficiency of the preset basis dimension *k* = 4 was validated via gam.check; no adjustment of knot numbers was required when convergence tests returned non-significant results.

A custom overdispersion test function was developed to quantify extra variance in Tweedie GAM outputs. The overdispersion ratio was calculated as model deviance divided by residual degrees of freedom. Models with an overdispersion ratio > 1.2 were classified as significantly overdispersed. In addition to the treatment of zero catches, no two-stage hurdle model (separate zero and count sub-models) was adopted. Instead, the Tweedie compound Poisson distribution was used to jointly fit zero-enriched catch data without independent zero-inflation stratification. During SI normalization, predicted values were constrained within the biologically reasonable range of [0,1]; corresponding 95% confidence intervals were synchronously truncated between 0 and 1 to avoid out-of-bound suitability values.

Boosted Regression Tree (BRT) models were implemented via the gbm.step function from the R dismo library, with 10-fold cross-validation (CV) to select the optimal number of regression trees. Fixed hyperparameters were specified as follows: tree complexity = 5 (allowing up to 5-way predictor interactions per tree), learning rate = 0.001 (adjusted to 0.005 for non-convergent Poisson submodels), and bag fraction = 0.6 (each tree trained on a random 60% subset of the full dataset). BRT models were used to quantify the relative importance of each environmental variable to the abundance, and these importance values were adopted as weights for calculating the Habitat Suitability Index (HSI). The gbm.step function automatically generated 3 core outputs: optimal total tree count (n.trees) corresponding to the minimum cross-validation deviance; cross-validation correlation coefficient, training and cross-validation deviance, and mean squared error (MSE); and raw relative variable influence values (rel.inf). Post-processing was conducted to retain only the five primary environmental predictors and implement secondary normalization:

(2) $$W_{i}=\frac{rel.{inf}_{i}}{\sum rel.{inf}_{env}}\;$$

Normalized weights summing to 1 were generated for each environmental covariate, which were further applied to weight and stack single-factor SI values for composite HSI calculation. GAM-derived uncertainty was calculated as: 95% parametric confidence bands around SI response curves quantified the uncertainty of suitability responses to single environmental gradients. BRT-derived uncertainty was calculated as follows: 10-fold cross-validation mitigated model overfitting, with cross-validation error reflecting overall predictive uncertainty of the BRT framework. Composite HSI uncertainty was calculated as follows: uncertainty bounds of integrated habitat suitability were linearly propagated from the confidence intervals of each individual environmental SI.

Based on the Arithmetic Mean Model (AMM), the composite Habitat Suitability Index (HSI) was calculated as the weighted sum of the single-factor Suitability Index (SI) and corresponding variable weights: *HSI* = $\sum_{i\;=\;1}^{n}\left({SI}_{i}\times {Weight}_{i}\right)$, where *SI_i_* represents the Suitability Index of the *i*-th environmental factor, Weight_i_ denotes its corresponding weight, and *n* is the total number of environmental variables. Habitat quality was stratified into three discrete tiers based on composite HSI scores: highly suitable habitat (HSI ≥ 0.7), moderately suitable habitat (0.4 ≤ HSI < 0.7), and unsuitable habitat (HSI < 0.4). A HSI validity test was implemented to verify model reliability: HSI values were grouped at 0.1 intervals, and the occurrence frequency (probability of presence) of target species within each interval was calculated to analyze the correlation between HSI and species distribution. All data cleaning, model construction, index computation, statistical analyses and visualization were performed in R (version 4.5.2).

## 3. Results

### 3.1. Seasonal Spatial Variations in C. bimaculata at Crab Sampling Stations

**Figure 2:**
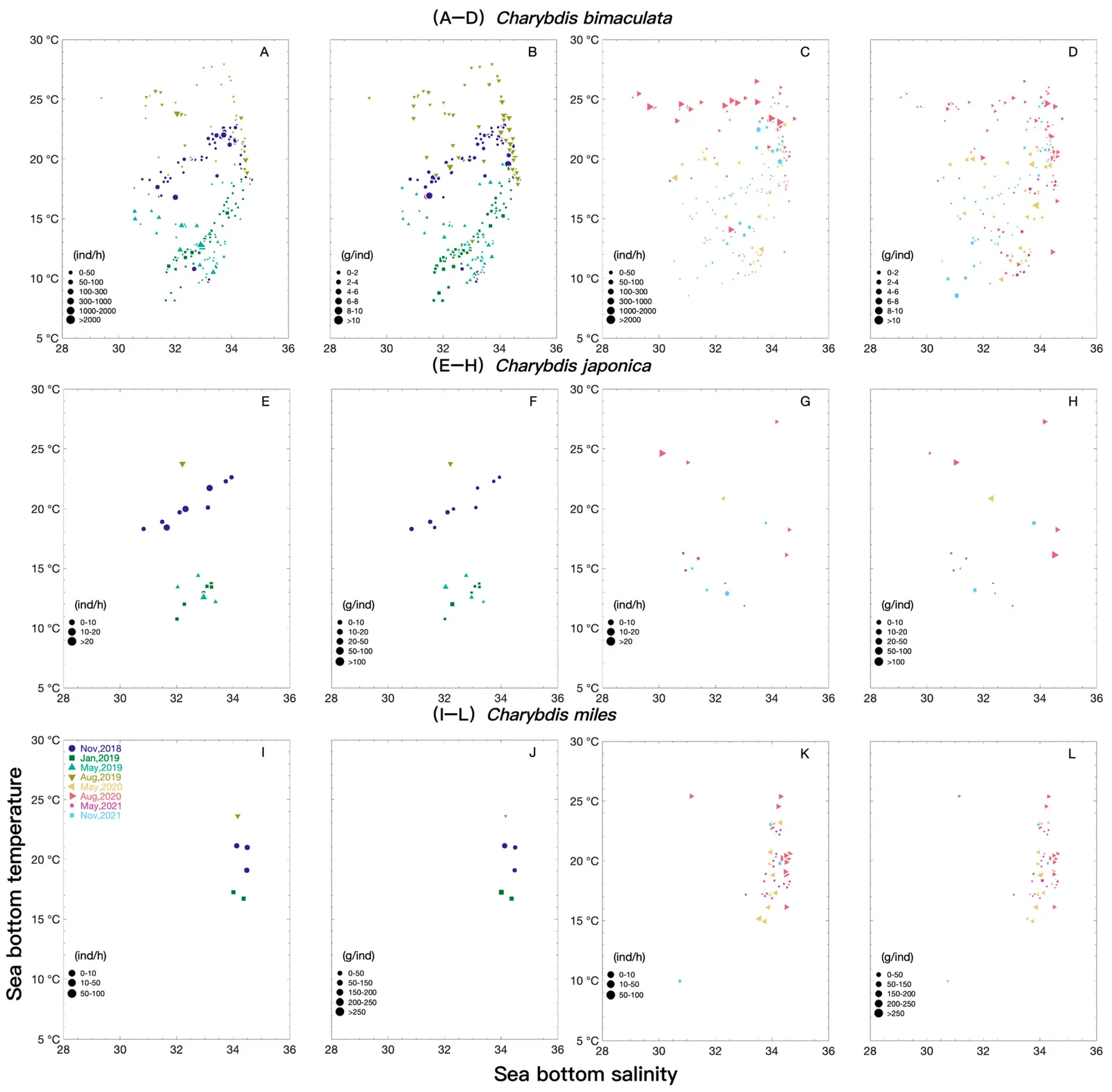
Associations between sea bottom salinity and sea bottom temperature (°C) with respect to two biological metrics of *Charybdis bimaculata*: numerical catch per unit effort (CPUE_n_), binned into the intervals 0–50, 50–100, 100–300, 300–1000, 1000–2000, and >2000 ind·h^−1^ in 2018–2019 (**A**); average individual weight (AIW), grouped into the categories (0–2, 2–4, 4–6, 6–8, 8–10, and >10 g·ind^−1^) in 2018–2019 (**B**); CPUE_n_ in 2020–2021 (**C**); and AIW in 2020–2021 (**D**). *C. japonica*: CPUE_n_ (0–10, 10–20 and >20 ind·h^−1^) in 2018–2019 (**E**), AIW (0–10, 10–20, 20–50, 50–100, and >100 g·ind^−1^) in 2018–2019 (**F**), CPUE_n_ in 2020–2021 (**G**), and AIW in 2020–2021 (**H**). *C. miles*: CPUE_n_ (0–10, 10–50, and 50–100 ind·h^−1^) (**I**), AIW (0–50, 50–150, 150–200, 200–250, >250 g·ind^−1^) (**J**), CPUE_n_ in 2020–2021 (**K**), and AIW in 2020–2021 (**L**). Seasonal data points are represented by colored circles: dark purple (November 2018), dark green (January 2019), teal (May 2019), olive gold (August 2019), pale cream yellow (May 2020), brick red (August 2020), magenta purple (May 2021), light sky blue (November 2021).

Across all sampling seasons, *C. bimaculata* occupied depths ranging from 10 to 115 m, with seabed salinity spanning 29–35 (Table 1). The minimum seabed temperature supporting species presence was recorded at 8 °C for *C. bimaculata* (Table 1). Most abundances (>100 ind·h^−1^) were found at SBTs of 17–23 °C, SBSs of 31–34, and depths of 10–70 m in November 2018; 11–16 °C, 32–34, and 40–70 m in January 2019; 10–16 °C, 30–34, and 10–70 m in May 2019; 18–26 °C, 31–35, and 20–100 m in September 2019; 12–23 °C, 30–35, and 10–80 m in May 2020; 21–27 °C, 29–35, and 10–70 m in August 2020; 18–19 °C, 30–31, and 20–30 m in May 2021; and 13–23 °C, 32–35, and 50–80 m in November 2021 (Figure 2). Individuals (<1 g·ind^−1^) were found at SBTs of 18–23 °C, SBSs of 31–35, and depths of 10–120 m in November 2018; 12–18 °C, 32–35, and 40–90 m in January 2019; 13–15 °C, 32–34, and 20–70 m in May 2019; 14–19 °C, 32–34, and 50–70 m in May 2020; 18–26 °C, 29–35, and 10–80 m in August 2020; and 13–19 °C, 32–34, and 50–70 m in November 2021 (Figure 2). This species preferred warm bottom water; highly suitable conditions included SST below 25 °C, high-salinity bottom environments, and a habitat depth range of 0–60 m (Figure S1).

In spring, *C. bimaculata* was concentrated in the Dasha–Lvsi fishing grounds (2019: SBT 10–16 °C, SBS 30–34, depth 13–70 m; 2020: 11–20 °C, 30–34, 13–74 m; 2021: 12–19 °C, 30–34, 24–53 m) and the East China Sea (2019: 11–20 °C, 30–35, 22–103 m; 2020: 13–23 °C, 31–35, 10–90 m; 2021: 14–19 °C, 32–35, 27–82 m). In summer, it was concentrated in the Dasha fishing grounds (2019: 18–26 °C, 29–32, 23–45 m; 2020: 11–26 °C, 29–34, 17–67 m) and the Yangtze River Estuary–Jiangwai–Zhoushan fishing grounds (2019: 13–25 °C, 31–35, 18–77 m; 2020: 21–25 °C, 31–35, 18–78 m). In autumn, the species was concentrated in Dasha–Lvsi (2018: 10–20 °C, 31–34, 14–76 m; 2021: 8–19 °C, 30–34, 14–78 m) and Zhoushan–Zhouwai–Yushan (2018: 18–23 °C, 33–35, 50–102 m; 2021: 17–24 °C, 32–35, 40–90 m). In winter, it was concentrated in the southern Yellow Sea (8–14 °C, 31–33, and 11–75 m) and the Zhoushan–Zhouwai–Yushan fishing grounds (14–20 °C, 33–35, and 41–114 m) (Table S1).

**Figure 3:**
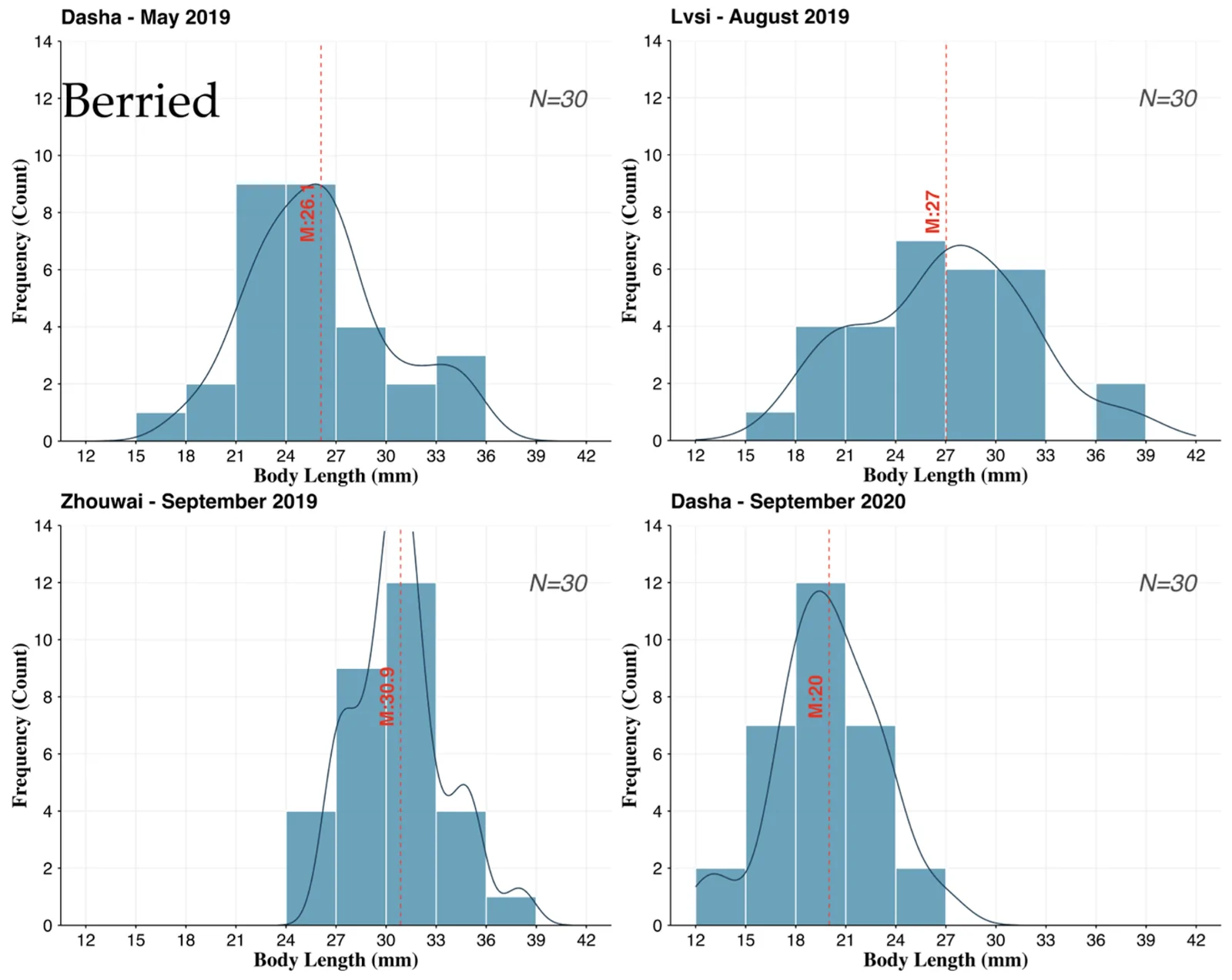
Length–frequency distributions of female crabs of *Charybdis bimaculata* collected at four sampling events covering multiple locations (Dasha, Lvsi, Zhouwai) from 2019 to 2020. Each panel presents the count of individuals across 12–42 mm carapace length intervals. Sampling location, month, year and sample size (N) are indicated above each histogram. The label “Berried” marks the presence of ovigerous females within the corresponding sample batch.

The seasonal order of total and mean CPUE_n_ of *C. bimaculata* was as follows: August 2020 > May 2019 and November 2021 (~50% of August 2020) > May 2020 (~25%) > November 2018, January 2019, and August 2019 (10–20%) > May 2021 (5%) (Table 2 and Table 3). The seasonal order of mean AIW was August 2019 and May 2021 > other survey seasons, indicating broodstock individuals in spring and summer (Table 3). At Dasha, two size groups of 12–21 mm and 27–36 mm were observed in January 2024; in May 2019, the peak fell within 21–27 mm. The above results indicate that small-sized individuals dominated in winter, while the average carapace length in late spring was markedly larger than that in winter. At Zhoushan in August 2023, the average carapace length reached a maximum value of 32.2 mm across sampling stations, with the primary peak at 30–33 mm (Figure 1, Figure 3 and Figure S2).

Individual body sizes were consistently smaller in winter, with a mean carapace length of 23–25 mm. Mean carapace length increased substantially during spring, ranging from 26 to 29 mm. Summer hosted the largest mature reproductive specimens; the maximum average length of 32.2 mm was recorded at the Zhoushan fishing grounds, identifying summer as the primary reproductive peak for large-bodied adults. In autumn, large-sized individuals were abundant at Zhouwai. Spawning grounds were identified at Dasha and the Zhoushan–Yushan–Wentai sea areas. Individuals measuring 27–33 mm constituted the dominant reproductive cohort across most sea areas and seasons (Figure 1 and Figure S2).

**Figure 4:**
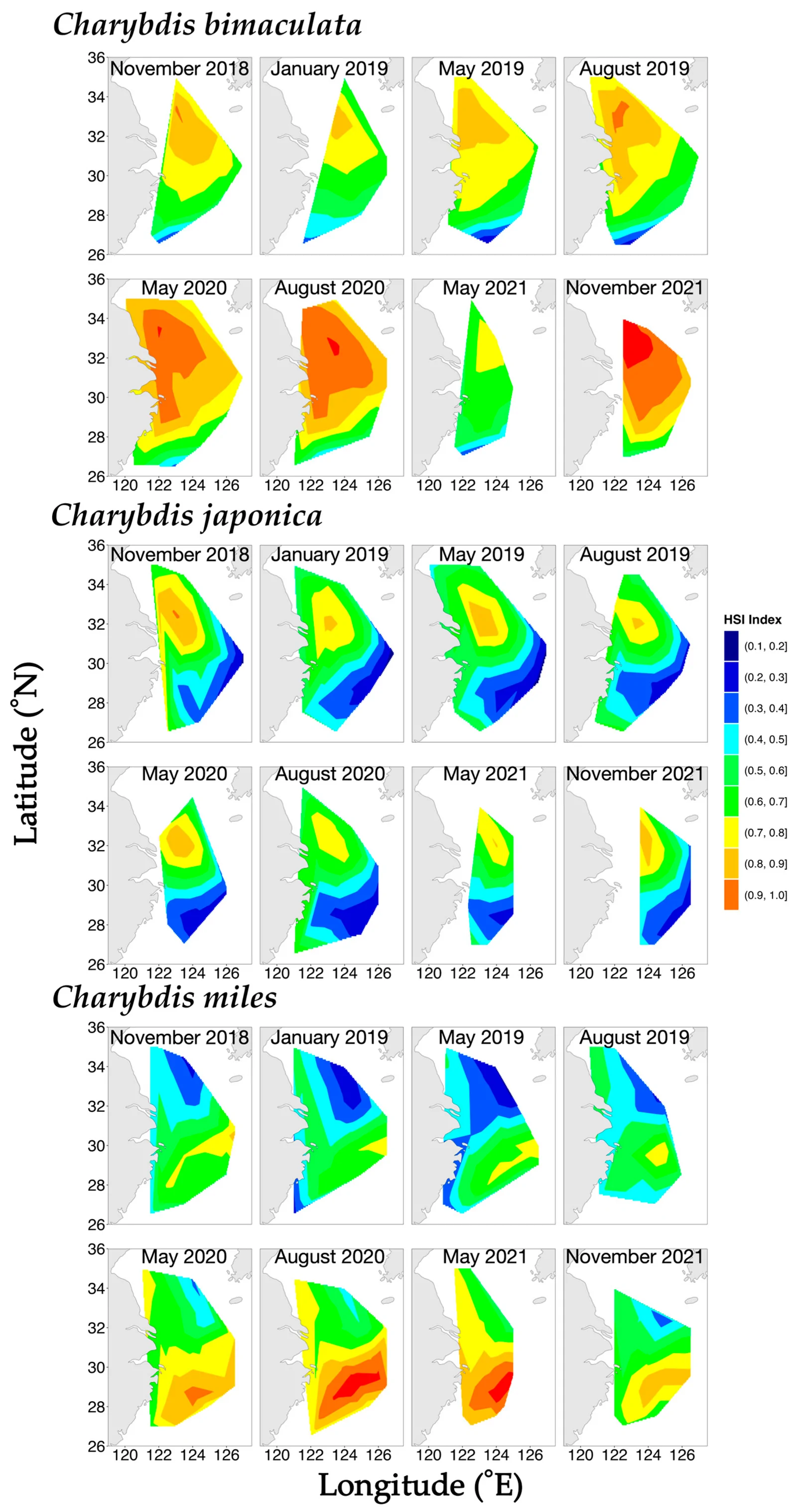
Spatiotemporal variation in the Habitat Suitability Index (HSI) of *Charybdis bimaculata*, *C. japonica*, and *C. miles* during different survey periods. The color scale denotes HSI values, where values closer to 1 represent optimal habitat conditions.

In November 2018 (autumn), the core highly suitable habitats of *C. bimaculata* were concentrated in 122–124° E and 32–34° N (the Dasha sea area off the coast of Jiangsu). The extent of highly suitable areas in November 2021 was comparable to that in November 2018. In January 2019 (winter), suitable habitats shrank, and highly suitable areas were restricted to the range of 32–34° N only. In May 2019 (spring), the area of highly suitable habitats increased markedly compared with January. In May 2020, the deep-red habitats were larger and more contiguous than those in May 2019, and highly suitable areas stretched westward close to the Yangtze River Estuary (around 122° E). In August 2019 (summer), the area of deep-red patches was larger than that in May, representing one of the periods with the broadest suitable habitat range throughout the year (Figure 4 and Figure S3).

Overall, *Charybdis bimaculata* was persistently concentrated in the waters off the Yangtze River Estuary to offshore Jiangsu (30–34° N, 122–125° E), while highly suitable habitats rarely occurred in the southern region of 26–29° N. Areas with high HSI values were consistently close to the continental coastline, and habitat suitability declined continuously in offshore waters east of 125° E. Summer (August) featured the broadest extent of suitable habitats and the highest HSI peaks across the whole year, with contiguous expansion of core habitats. Spring (May) brought moderately high suitability yet exhibited intense interannual fluctuations; conditions were highly suitable in 2020 but extremely poor in 2021. Highly suitable habitats contracted in autumn (November), though stable core patches remained. Winter (January) saw the narrowest suitable range of the year (Figure 4 and Figure S3).

### 3.2. Seasonal Spatial Variations in C. japonica at Crab Sampling Stations

*Charybdis japonica* was found at a depth range of 16–104 m, predominantly in inshore shallow waters (Table 1). The SBT value ranges were 10–16 °C in spring and winter, and 16–27 °C in summer and autumn (Table 1). Individuals (<10 g·ind^−1^) of *C. japonica* were found at SBTs of 18–23 °C, SBSs of 31–34, and depths of 30–70 m in November 2018; 10–14 °C, 32–34, and 30–50 m in January 2019; and 12–15 °C, 31–32, and 40–70 m in November 2021 (Figure 2). *Charybdis japonica* typically favored low salinity and low temperature; its highly suitable habitats were estuarine and nearshore waters with low SSS; suitability decreased gradually in high-salinity offshore waters (Figure S1). The seasonal order of total CPUE_n_ of *C. japonica* was November 2018 and May 2021 > August 2020 (60% of May 2021) > January 2019, May 2019, and November 2021 (50%) > August 2019 (20%) > May 2020 (5%) (Table 2). The maximum AIW showed a seasonal order of August 2020 and November 2021 > November 2018, January 2019, May 2019, and May 2021, indicating that some individuals could grow larger in autumn and winter (Table 3).

*Charybdis japonica* was concentrated in the Dasha–Yangtze River Estuary in spring (2019: SBT 12–15 °C, SBS 32–34, depth 28–50 m; 2020: 21 °C, 32, 27 m; 2021: 12–16 °C, 30–33, 22–42 m) (Table S1). In summer, *C. japonica* inhabited the Yangtze River Estuary. In the Yangtze River Estuary, there was mainly an aggregation area for juveniles, characterized by high individual numbers but low biomass, while large-sized individuals remained in Dasha for nursery, and some of the juveniles dispersed to Zhouwai in autumn and winter (Table S1). In November 2018 (autumn), the highly suitable red core habitat zone of *C. japonica* was distributed between 32–34° N and 122–124° E (Figure 4 and Figure S3). The offshore southern waters (26–29° N, east of 124° E) were entirely unsuitable for *C. japonica*. Autumn represents the period with the highest habitat suitability for *C. japonica* (Figure 4 and Figure S3). In January 2019 (winter), water temperatures dropped, and the overall habitat suitability contracted (Figure 4 and Figure S3).

In terms of interannual variations, autumn generated the most favorable conditions, with extensive red prime suitable zones observed in both 2018 and 2021; suitable habitats shrank drastically in winter. Moderately suitability prevailed in spring and summer, and habitat suitability for *C. japonica* decreased rather than increased as water temperatures rose in summer (Figure 4 and Figure S3).

### 3.3. Seasonal Spatial Variations in C. miles at Crab Sampling Stations

The lowest observed water temperature associated with the occurrence of *C. miles* in in the study area during this survey was not less than 10 °C (Table 1). *Charybdis miles* is a eurythermal species that can survive in both low-temperature and high-temperature waters (Figure S1). This species favors high-salinity offshore waters and can barely survive in low-salinity estuarine areas (Figure S1). Habitat suitability increased with water depth, and this crab was rarely distributed in shallow waters (Figure S1).

*Charybdis miles* occurred in Zhoushan–Zhouwai–Yushan in spring (2020: SBT 15–24 °C, SBS 33–35, depth 40–80 m; 2021: 17–23 °C, 33–35, 27–85 m), Zhouwai–Yuwai (2020: 18–25 °C, 34–35, 64–95 m) in summer, Dasha–Wentai (2021: 10–23 °C, 31–34, 23–70 m) in autumn, and Yushan (2019: 16–18 °C, 34–35, 78–92 m) in winter (Table S1). The seasonal order of total CPUE_w_ and CPUE_n_ of *C. miles* was May 2021 > August 2020 (60% of May 2021) > May 2020 (36%) > November 2018, January 2019, August 2019, and November 2021 (<10%) (Table 2). The mean AIW showed a seasonal pattern: November 2018 and January 2019 > May 2020 and 2021 > August 2019 and 2020 and November 2021 (Table 3).

During May (spring) of 2019, 2020 and 2021, contiguous orange zones of high suitability emerged within 28–30° N and 123–126° E. In 2020, yellow and orange suitable belts extended northward to 31° N, and in 2021, yellow highly suitable zones stretched as far north as 33° N. In August 2020, the highly suitable belt shifted westward close to 122° E, covering a latitudinal range of 27–31° N. This August survey recorded the most favorable habitat conditions for *C. miles* across the 2018–2020 study period. In November 2018 and 2021 (autumn), small highly suitable habitats were exclusively distributed in the offshore southeastern waters spanning 28–30° N and 124–126° E, while suitability remained extremely low in all waters north of 32° N. In January 2019 (winter), the range of highly suitable habitats contracted further (Figure 4 and Figure S3).

### 3.4. Statistical Analysis Among Years and Seasons

The Kruskal–Wallis nonparametric test was applied to station-level CPUE_n_ data (including zero catches) to examine interannual variations from 2018 to 2021. Sample sizes were n = 127 for 2018, n = 401 for 2019, n = 283 for 2020, and n = 192 for 2021. No significant overall interannual difference was detected for *C. bimaculata* during 2018–2021 (*χ*^2^ = 2.443, *df* = 3, *p* = 0.486), while highly significant interannual differences were observed for *C. miles* (*χ*^2^ = 49.763, *df* = 3, *p* = 8.97 × 10^−11^ < 0.05) and *C. japonica* (*χ*^2^ = 7.961, *df* = 3, *p* = 0.046 < 0.05). Post hoc pairwise multiple comparisons with Bonferroni-corrected Dunn’s test revealed no statistically significant differences between any pair of years for *C. bimaculata* and *C. japonica* (*p* > 0.05). This apparent inconsistency between the marginally significant global Kruskal–Wallis result and non-significant pairwise contrasts for *C. japonica* can be attributed to the strict correction criterion of the Bonferroni method. With four year groups, six pairwise comparisons were conducted, which reduced the effective per-test significance threshold to α = 0.05/6 ≈ 0.0083. The weak interannual effect sizes only produced a borderline significant global signal but failed to satisfy the far stricter threshold for individual year-to-year comparisons after multiple-testing adjustment.

Significant seasonal differences in CPUE_n_ were detected for *C. bimaculata* (*χ*^2^ = 10.6937, *df* = 3, *p* = 0.01), extremely significant seasonal differences for *C. miles* (*χ*^2^ = 14.977, *df* = 3, *p* = 0.00184), and no significant seasonal differences for *C. japonica* (*χ*^2^ = 6.235, *df* = 3, *p* = 0.101). For *C. bimaculata*, a significant difference was only found between spring and autumn (*Z* = 2.884, *df* = 3, *p* = 0.0118). For *C. miles*, significant differences existed between spring and autumn (*Z* = −3.292, *df* = 3, *p* = 0.003), as well as spring and winter (*Z* = 2.921, *df* = 3, *p* = 0.0105). Sample sizes were n = 374 for spring, n = 282 for summer, n = 232 for autumn, and n = 115 for winter.

### 3.5. Verification of Model Reliability Based on Residual Distributions and HSI Density

The scatter plots of residuals against linear predictors for each environmental variable (SST, SBT, SSS, SBS, depth) exhibited no obvious systematic curved trends and showed evenly scattered distributions, indicating that the model captured the nonlinear relationships adequately with reasonable fitted trends (Figure S4). Most points in the residual Q-Q theoretical quantile plots aligned closely with the reference line, with only a small number of extreme outliers deviating from it; the residuals generally approximately satisfied the normality assumption (Figure S4). The response-versus-fitted plots and residual histograms revealed that residuals clustered around zero, with no evident heteroscedasticity where variance expanded or contracted alongside fitted values (Figure S4). The residual distribution remained stable, supporting reasonable internal fitting performance of the model, though the lack of independent external validation may lead to underestimated prediction uncertainty and inflated overall model reliability (Figure S4). Species occurrence showed a significant correlation with HSI values, with clear demarcation between samples from highly suitable habitats and less suitable habitats (Figure S5).

Collectively, internal residual diagnostics and within-dataset HSI–species occurrence correlations only offer preliminary evidence that seasonal and year effects may be partially distinguishable within our sampled dataset. Nevertheless, this conclusion is subject to critical constraints stemming from our highly unbalanced temporal sampling design: winter sampling is limited solely to January 2019, autumn surveys only cover November 2018 and November 2021–January 2022, summer data were collected in 2019 and 2020, and spring samplings span 2019 to 2021. Standard residual diagnostics merely describe residual distribution characteristics and cannot fully eliminate confounding signals between seasonal variability and interannual fluctuations. Additionally, all these assessments rely entirely on the identical dataset used for model fitting, leading to circular validation without reserved independent spatial or temporal subsets for external testing; this dual limitation may underestimate genuine prediction uncertainty and inflate the apparent predictive performance of the HSI model for unsampled sea areas. All internal diagnostics below rely on training data only; key limitations related to absent external validation are elaborated in Section 4.4, “Limitations”.

## 4. Discussion

### 4.1. CPUE_w_ and CPUE_n_ Variations in C. bimaculata, C. japonica, and C. miles

The absolute abundance of *C. bimaculata* presented in this study was consistently higher than that of *C. japonica* and *C. miles* across the seasons in the study area. Both CPUE_w_ and CPUE_n_ of *C. bimaculata* peaked in May and were lowest in January. But the biomass was relatively high in winter in Haizhou Bay [17]. In Ise Bay, the abundance and biomass of this crab decreased in summer, and then increased through new recruitment from autumn (October–November) to the following spring (March–May) [18]. The biomass in the Zhejiang coastal water area exhibited a progressive monthly increase from April to June [19].

The resource index (CPUE_w_ and CPUE_n_) and AIW of *C. japonica* presented in this study peaked in August, immediately after the summer fishing moratorium, and then gradually declined in autumn and winter due to heavy fishing pressure; in spring, with increasing water temperature, the stock began to grow, leading to a recovery in CPUE_w_, CPUE_n_, and AIW. In the East China Sea, Yu et al. (2005) found that CPUE_w_ and CPUE_n_ followed the seasonal order of November > August > May > February, and the widest distribution and the highest resource density were observed in January [20].

The CPUE_n_ of *C. miles* presented in this study decreased continuously after the summer fishing moratorium. The abundance of juvenile crabs began to increase in July–August, peaked in November, and remained high through February of the following year [8]. In the East China Sea of 1998–1999, the seasonal order was February (298.3 g·h^–1^) > November (217 g·h^–1^) > May (137.9 g·h^–1^) > August (131.9 g·h^–1^) [8].

The current summer fishing moratorium in China is implemented annually from May 1 to September 16. Ovigerous female *C. bimaculata* individuals have been documented in both the southern Yellow Sea and the East China Sea from April to May (Figure S2), indicating that this species likely underwent a major spawning peak prior to the summer fishing moratorium. Accordingly, the CPUE_w_ of *C. bimaculata* reached a minor peak in May, followed by a sharp decline as mature spawners died post-spawning. Meanwhile, datasets for *C. japonica* and *C. miles* demonstrated that their CPUE_w_ dropped rapidly after the moratorium period. Dramatic shifts occurred in the seasonal patterns of both CPUE_w_ and CPUE_n_ for these three crab species between 1998 and 2018.

### 4.2. Issue About Spawning Periods of Three Crab Species

The spawning season of *C. bimaculata* in the study area was inferred to be from April to May and from August to September, suggesting that the species can spawn in spring and summer, and small juveniles can be observed and continuously recruited in autumn and winter (Figure S4). Zheng (2015) found that *C. bimaculata* had a relatively long spawning period, and ovigerous females can be caught in spring, summer, and autumn in the Yellow and East China Seas [5]. In Tokyo Bay, berried females were collected from spring to autumn; most individuals contributed to spawning one year post hatch and then died by the winter of the same year [21]. In Kagoshima Bay, the spawning season was suggested to be from May to November, with the peak in July–August, and hatching occurring from June to October [22].

*Charybdis japonica* generally reproduces in summer and spawns twice a year, from April to May and August to September in Zhejiang [5]. *Charybdis japonica* spawns from June to July along the coast of Shandong [5]. In Fukuoka, Japan, the reproductive season of *C. japonica* is limited to late May to early October, and their gonads regress during other seasons [23]. Approximately one-year-old crabs start reproducing after July and spawn at least twice until September [23]. In Prymorye, Russia, and the northwestern area of the Japan Sea, the breeding season lasts from June to September [24]. In the Seto Inland Sea of Japan, most adults already start reproduction in May [25]. Additionally, the spawning period of *C. miles* lasts from May to December, with the peak from August to October [8].

In summary, within our study area, the spawning seasons of the three crab species differ distinctly: ovigerous *C. bimaculata* spawns from April to May and August to September; *C. japonica* spawns from April to September; and *C. miles* spawns from May to December.

### 4.3. Issue About Suitable Environment and Habitat of Three Crab Species

In the Yellow and East China Seas, the highest survival rate of *C. bimaculata* was obtained at a salinity of 35 and 24 °C [26]. In Haizhou Bay located in the northern Yellow Sea, depth and SSS had significant effects on the resource distribution of *C. bimaculata*, and both showed a positive correlation with the relative abundance [17]. In the coastal waters of southern Zhejiang, *C. bimaculata* mainly occurred in waters with SSTs of 11–13 °C and SSSs of 30–32 in April; SSTs of 16–20 °C and SSSs of 32–35 in May; and SSTs of 19–21 °C and SSSs of 31–34 in June [19]. In this study, most abundances were found at SSTs of 12–25 °C, SSSs of 22–35, SBT 10–23 °C, SBSs of 30–35, and depths of 10–80 m, and juveniles were found at SSTs of 14–22 °C, SSSs of 31–34, SBT 13–19 °C, SBSs of 32–34, and depths of 20–70 m in May. Xue et al. (2024) predicted that increased SST caused by climate change enabled the invasion of *C. bimaculata* from the northern Yellow Sea waters into the southern Bohai Sea, where it can overwinter and complete its life cycle [27]. In this study, in both spring and summer of 2020, the HSI values reached their maximum for the corresponding seasons. The suitable habitats displayed an obvious seasonal pattern of northward expansion and southward contraction: habitats expand northward and toward offshore waters in summer, yet retreat to inshore areas in winter (Figure 3).

The survival water temperature of *C. japonica* was 3–32 °C, with an optimum growth temperature of 20–27 °C. The water temperature for spawning of the female ranged from 20 to 28 °C, and they could spawn several times per year; the reproductive season was limited to water temperatures of >18–20 °C [28]. *Charybdis japonica* preferred a warm water temperature and SBS of 29–31, with a low biomass at 10–13 °C [29]. Thus, this study reveals that August to November constitutes the highly suitable window for their reproduction and somatic growth. Juveniles were found at SBTs of 12–23 °C, SBSs of 31–34, and depths of 30–70 m in November, and 10–14 °C, 32–34, and 30–50 m in January. Previous studies have shown that in the East China Sea, *C. japonica* was mainly distributed in Dasha and the Yangtze River Estuary at 20–60 m depths (north of 31° N), as well as around coastal islands and reefs at 10–30 m depths in the central and southern East China Sea [20]. Resource density was generally higher in inshore areas than in offshore areas, showing a trend of increased resource density closer to the coast and toward the north, and high-biomass areas were found in the Yangtze River Estuary, Dasha, and Zhoushan at water depths of 20–60 m [16]. In this study, warm spring and summer conditions suppressed the habitat suitability of *C. japonica*, and its populations contracted toward shallow coastal waters in summer. The southeastern offshore waters remained unsuitable year-round and were not key distribution areas for *C. japonica*.

Generally, *C. miles* prefers high-salinity, deep offshore waters and cannot tolerate low-salinity diluted coastal waters, showing an entirely opposite salinity preference to *C. japonica* (Figure S3). No obvious seasonal variation in *C. miles* could be detected, with the distribution across almost the entire survey area, showing poor aggregation and no distinct central fishing grounds in this study. *Charybdis miles* was distributed in the southern East China Sea (i.e., the central and southern East China Sea), occurring in waters south of 31° N at depths of 40–100 m [8].

Ecologically, *C. bimaculata* favors shallow seas with warm bottom water and euryhaline conditions; *C. japonica* prefers low-salinity coastal waters with cold bottom water; and *C. miles* thrives in deep, high-salinity offshore waters with eurythermal tolerance. Overall, the core habitats of *C. bimaculata* and *C. japonica* lie in the northern offshore waters outside the Yangtze River Estuary (32–34° N). The suitable habitat area of *C. miles* expands greatly in spring and summer yet exhibits dramatic interannual fluctuations, whereas low temperatures in autumn and winter drastically shrink the habitat range of *C. miles* (Figure 3).

### 4.4. Fisheries Management Issues, Study Limitations, and Future Works

Since 2004, China’s government has implemented a minimum landing size of 50 mm carapace width, and individuals of *C. japonica* with a width < 50 mm have been prohibited from sale [20]. In addition, in 2014, the local government in Shandong issued a local regulation stating that the bycatch ratio of undersized crabs should not exceed 25% of the total crab catches [30]. To protect crab juveniles from overfishing, the local government in Zhejiang issued a local standard for the allowable size of capture and juvenile proportion of key marine crab species in 2014 [30]. The standard stipulates a minimum landing size of 60 mm for swimming crab and that the proportion of young crab should not exceed 20% of the total catch [30]. Although these measures can help the recovery of recruitment and spawning stocks to achieve maximum sustainable yield for both biological and economic objectives, amending conservation measures for specific species or species groups remains challenging.

A major analytical limitation of our HSI modeling workflow stems from our single-dataset validation scheme. All diagnostic assessments—including residual distribution checks and correlations between HSI scores and species occurrence density—were conducted using the same dataset used to calibrate Tweedie GAM and BRT models; no geographically or temporally independent subsets were reserved for external out-of-sample validation. This circular validation approach inherently underestimates predictive error and overinflates apparent model performance, as internal diagnostics cannot quantify predictive robustness across unsampled spatial domains or unmonitored survey years. No quantitative predictive metrics (e.g., AUC, explained deviance, out-of-sample correlation) from external testing are available to rigorously benchmark model performance, meaning all interpretations of species habitat segregation, seasonal habitat expansion/contraction, and core suitable grounds carry unquantified predictive uncertainty. Additionally, uneven seasonal sampling effort across survey years exacerbates this issue: interannual comparisons of habitat suitability may be biased by imbalanced station coverage, and spatial autocorrelation within trawl samples further inflates apparent model fit without independent cross-checks. Consequently, all ecological inferences derived from HSI outputs—including distinct niche separation among *Charybdis* species and seasonal shifts in suitable habitats—should be interpreted cautiously, as the true magnitude of prediction uncertainty cannot be constrained with our current modeling workflow. Claims of strong or high model reliability are not fully supported without independent out-of-sample validation.

To resolve the validation constraints outlined above and improve the robustness of crab habitat predictions, four targeted improvements will be implemented in follow-up research: (1) Multi-layer cross-validation design: Integrate spatial block cross-validation (partitioning the study area into disjoint spatial grids) and stratified temporal cross-validation (split datasets by survey year) to separate training and testing subsets without geographic or temporal overlap. (2) Independent external validation dataset: Reserve 20% of trawl stations as a fully isolated out-of-sample dataset prior to model fitting, constructing a three-stage evaluation pipeline (training set → internal cross-validation set → independent external validation set) to quantify real predictive error. (3) Supplementary quantitative performance metrics: Calculate standardized diagnostic indicators including proportion of explained deviance, Pearson correlation between predicted HSIs and field CPUE, binary AUC from presence–absence classification based on abundance thresholds, and continuous spatial uncertainty surfaces to quantify prediction bias across the entire study domain. (4) Multi-model comparative optimization: Test alternative smooth term parameters, weighted HSI calculation schemes, and zero-inflated BRT architectures to reduce subjective choices during model configuration, and compare predictive performance across multiple SDM algorithms to strengthen the generalizability of habitat suitability outputs. Beyond validation improvements, follow-up work could also incorporate additional environmental covariates (e.g., sediment type, chlorophyll-a, tidal current velocity) to refine niche quantification for the three co-occurring *Charybdis* crabs and support spatially differentiated seasonal fishery management measures.

## 5. Conclusions

Habitat suitability was modeled from survey CPUE and environmental associations and the results should be interpreted within the limits of unbalanced seasonal sampling and non-independent HSI validation. The main conclusions are summarized as follows:

(1) The highest CPUE_n_ values of *C. bimaculata*, *C. japonica* and *C. miles* were found during August 2020, November 2018, and May 2021 in the study area.
(2) The spawning period of *C. bimaculata* is suggested to be April–May and August–September, with the spawning grounds covering Dasha and waters south of the Yangtze River Estuary.
(3) *Charybdis japonica* and *C. bimaculata* concentrate in the Yangtze River Estuary, and *C. miles* is distributed sparsely in the southern East China Sea.
(4) Within the bounds of our model predictions (which carry uncertainty inflated by the absence of independent external validation), habitat suitability peaks of the three swimming crab species appear fully spatially segregated across the study domain.

## Figures and Tables

**Table 1:** Seasonal data ranges of environmental factors including sea surface temperature (SST), sea surface salinity (SSS), sea bottom temperature (SBT), sea bottom salinity (SBS), and depth for the crab species *Charybdis bimaculata*, *C. japonica*, and *C. miles* in 2018–2021.

Survey Time	SST	SBT	SSS	SBS	Depth
	*C. bimaculata*				
November, 2018 (autumn)	17–25	10–23	30–35	30–35	14–115
January, 2019 (winter)	8–19	8–19	31–35	31–35	11–114
May, 2019 (spring)	12–20	10–20	29–34	30–35	13–103
September, 2019 (summer)	25–30	13–28	16–34	29–35	17–104
May, 2020 (spring)	12–25	10–23	26–35	30–35	10–86
August, 2020 (summer)	24–30	10–27	21–35	29–35	14–112
May, 2021 (spring)	15–25	14–18	28–34	32–35	26–82
November, 2021 (autumn)	8–26	8–25	29–34	30–35	14–89
	*C. japonica*				
November, 2018 (autumn)	18–23	18–23	31–34	30–34	28–66
January, 2019 (winter)	11–14	10–14	32–34	32–34	33–48
May, 2019 (spring)	14–16	12–15	31–33	32–34	28–50
September, 2019 (summer)	28	24	24	32	28
May, 2020 (spring)	22	21	31	32	27
August, 2020 (summer)	24–30	16–27	25–35	30–35	16–104
May, 2021 (spring)	16–20	12–16	27–33	30–33	22–42
November, 2021 (autumn)	12–18	12–19	31–34	31–34	30–84
	*C. miles*				
November, 2018 (autumn)	22–24	19–21	34–35	34–35	73–100
January, 2019 (winter)	16–18	16–18	34–35	34–35	78–92
September, 2019 (summer)	29	24	34	35	70
May, 2020 (spring)	18–25	15–23	31–34	33–35	40–80
August, 2020 (summer)	25–30	16–26	29–35	31–35	7–104
May, 2021 (spring)	18–25	16–23	30–35	33–35	26–85
November, 2021 (autumn)	10–23	10–23	30–34	30–34	23–70

**Table 2:** Total CPUE_w_ and CPUE_n_ of the crab species *Charybdis bimaculata*, *C. japonica*, and *C. miles* across the 2018–2021 season.

Survey Season/Station Number	*Charybdis bimaculata*		*Charybdis japonica*		*Charybdis miles*	
	CPUE_w_	CPUE_n_	CPUE_w_	CPUE_n_	CPUE_w_	CPUE_n_
November, 2018/127	7052.3	3447.4	236.2	62.9	815.5	6
January, 2019/111	5506.8	1992.1	103.1	29.4	564.1	3
May, 2019/141	24,577	10,618.7	460.8	30.6	no catch	no catch
August, 2019/140	20,624.4	3201.9	271.2	12	188.1	3.8
May, 2020/143	18,640.1	5129.9	207.8	3.4	4325.2	66.9
August, 2020/140	40,502.7	20,832.8	1855.2	41.8	7819.3	111.1
May, 2021/87	5810	1130.5	1501	65.8	12,155.6	171.3
November, 2021/105	23,915	10,707	278.1	28.8	294	3.9

**Table 3:** Seasonal data for the mean value (±standard deviation) and value range of catch per unit effort in weight (CPUE_w_), catch per unit effort in number (CPUE_n_), and average individual weight (AIW) for *Charybdis bimaculata*, *C. japonica*, and *C. miles* in 2018 to 2021.

Survey Season	Mean CPUE_w_ at Collection Stations	Mean CPUE_n_ at Collection Stations	Mean AIW	Value Range of CPUE_w_	Value Range of CPUE_n_	Value Range of AIW
	*C. bimaculata*					
November, 2018	115.6 ± 188.1	56.5 ± 98	2.5 ± 2	0.3–1117	0.8–610	0.4–9.9
January, 2019	95 ± 157.6	34.4 ± 48.6	2.8 ± 1.7	2.4–932	1–296	0.5–9.8
May, 2019	481.9 ± 1200.2	208.2 ± 558.2	2.8 ± 1	1.5–7212.6	1–3579	0.7–5.4
August, 2019	420.9 ± 1168	65.3 ± 164.5	6.1 ± 7.8	4.6–6868.2	1–1047.3	2.1–58.7
May, 2020	358.5 ± 516.8	98.7 ± 161.3	4 ± 2.1	1.2–2920	1–1040	0.1–12.4
August, 2020	698.3 ± 1215.1	359.2 ± 687.2	3.3 ± 2.4	4.3–5528.9	0.9–3324	0.3–11
May, 2021	223.5 ± 427.2	43.5 ± 91	6.5 ± 3.8	2.8–1632	1–384	1.4–19
November, 2021	498.2 ± 1210.6	223.1 ± 581.9	2.8 ± 2.1	10.2–6640	4–3168	0.8–10.7
	*C. japonica*					
November, 2018	26.2 ± 24.2	7 ± 5.7	6.5 ± 6.2	5.2–84.5	1.2–18.8	1–17.4
January, 2019	17.2 ± 12.2	4.9 ± 3.4	7.2 ± 9.1	7.9–38.4	1–8	1.2–25
May, 2019	115.2 ± 87	7.6 ± 6.2	14.3 ± 9.6	3.8–211.2	2.1–16	1.9–24.8
August, 2019	271.2	12	22.6	not estimated	not estimated	not estimated
May, 2020	207.8	3.4	60.6	not estimated	not estimated	not estimated
August, 2020	371 ± 531.5	8.4 ± 8.4	60.7 ± 47.2	44.5–1313	1.5–22.3	2–131.3
May, 2021	300.2 ± 283.1	13.2 ± 10.5	19.5 ± 6.3	26–728	2–28	12.9–26
November, 2021	69.5 ± 64.9	7.2 ± 9.7	44.3 ± 48.5	13–150.5	1–21.6	0.6–95.2
	*Charybdis miles*					
November, 2018	271.8 ± 161.4	2 ± 1	134 ± 19.5	139–451.5	1–3	112.5–150.5
January, 2019	282 ± 84.5	1.5 ± 0.7	198.9 ± 39.5	222.3–341.8	1–2	170.9–226.8
August, 2019	188	3.8	49.5	not estimated	not estimated	not estimated
May, 2020	480.6 ± 298.1	7.4 ± 8	96 ± 57.1	50–974.5	0.8–26.7	29.8–203.4
August, 2020	558.5 ± 619.8	7.9 ± 9.5	79.6 ± 31.2	72.7–2319.5	1–33.2	43.5–166
May, 2021	506.5 ± 948.4	7.1 ± 17.9	99.3 ± 54.1	43–4794	0.9–90	10.8–262
November, 2021	98 ± 102.9	1.3 ± 0.6	63 ± 46.4	12.7–212.3	0.9–2	13.8–106.2

## Data Availability

The original contributions presented in this study are included in the article. Further inquiries can be directed to the corresponding authors.

## Supplementary Materials

The following supporting information can be downloaded at: https://www.mdpi.com/article/10.3390/biology15161406/s1, Supplementary File S1: **Table S1**: Mean and total values of catch per unit effort in weight (W), W%, catch per unit effort in number (N), N%, and average individual weight (AIW) for *Charybdis bimaculata*, *Charybdis japonica*, and *Charybdis miles* across survey season, fishing grounds, and environmental conditions such as sea surface temperature (SST), sea surface salinity (SSS), sea bottom temperature (SBT), sea bottom salinity (SBS), and depth in 2018–2021. **Figure S1**: Habitat suitability response curves of three *Charybdis* species (*C. bimaculata*, *C. japonica*, and *C. miles*) against key environmental variables, including sea surface temperature (SST), sea surface salinity (SSS), sea bottom temperature (SBT), sea bottom salinity (SBS), and depth. The y-axis represents habitat suitability values ranging from 0 to 1, while the x-axis indicates the gradient of each corresponding environmental factor. **Figure S2**: Length–frequency distributions of female crabs of *Charybdis bimaculata* collected at five sampling events covering multiple locations (Lianqingshi, Dasha, Zhoushan, Yushan, Wentai) from 2023 to 2026. Each panel presents the count of individuals across 12–42 mm carapace length intervals. Sampling location, month, year and sample size (*N*) are indicated above each histogram. The label “Berried” marks the presence of ovigerous females within the corresponding sample batch. **Figure S3**: Seasonal distribution patterns of CPUE_w_ (unit: g·h^−1^) for (A–H) *Charybdis (Gonioneptunus) bimaculata* (Miers, 1886), binned into the intervals 0–50, 50–100, 100–200, 200–500, 500–1000, 1000–3000, 3000–5000, and >5000 g·h^−1^; (I–P) *Charybdis (Charybdis) japonica* (A. Milne-Edwards, 1861), binned into the intervals 0–50, 50–100, 100–200, 200–300, and >300 g·h^−1^; and (Q–X) *Charybdis (Charybdis) miles* (De Haan, 1835), binned into the intervals 0–200, 200–400, 400–600, 600–800, 800–1500, and >1500 g·h^−1^. (A, I, Q) November 2018, (B, J, R) January 2019, (C, K, S) May 2019, (D, L, T) August 2019, (E, M, U) May 2020, (F, N, V) August 2020, (G, O, W) May 2021, (H, P, X) November 2021. **Figure S4**: Residual diagnostic plots for the Generalized Additive Model of environmental variables such as sea surface temperature, sea surface salinity, sea bottom temperature, sea bottom salinity and depth for crab species *Charybdis bimaculata*, *Charybdis japonica*, and *Charybdis miles*. Each group contains four subgraphs: deviance residuals against linear predictors, Q-Q normal quantile plots, response versus fitted values, and histograms of residuals. **Figure S5**: Habitat Suitability Index (HSI) density distributions for three *Charybdis* crab species: *Charybdis bimaculata*, *C. japonica*, and *C. miles*. Density curves are separated by observation status (presence vs. absence) to illustrate differences in HSI value frequency between sampling sites with and without target crab occurrence. Supplementary File S2: the code.
