# Supplementary material for: Spatiotemporal and Interannual Habitat Variability of Three Charybdis Swimming Crab Species (Brachyura: Portunidae) in the Southern Yellow Sea and East China Sea
Source: Biology (Basel). 2026 Aug 17;15(16):1406. doi: 10.3390/biology15161406 (PMC13509451; doi:10.3390/biology15161406)
Supplement: Supplementary file 1 [file biology-15-01406-s001.zip › Supplementary File S1.pdf]

Supplementary file S1

Table S1. Mean and total values of catch per unit effort in weight (W), W%, catch per unit effort in number (N), N%, and average individual weight (AIW) for *Charybdis bimaculata*, *Charybdis japonica*, and *Charybdis miles* across survey season, fishing ground, and environmental conditions such as sea surface temperature (SST), sea surface salinity (SSS), sea bottom temperature (SBT), sea bottom salinity (SBS), and depth in 2018–2021.

Fishing ground	Mean value					Total value				Environmental factor				
	W	W%	N	N%	AIW	W	W%	N	N%	SST	SSS	SBT	SBS	Depth
	Charybdis bimaculata													
	November, 2018 (Autumn)													
Lvsi	169.3	19.7%	82	18.3%	4.6	1354.7	19.2%	655.7	19%	17–19	31–32	17–19	31–32	14–33
Dasha	155.8	18.1%	49.7	11.1%	2.6	2180.6	30.9%	695.8	20.2%	18–22	30–33	10–20	31–34	28–76
Yangtze River Estuary	54.7	6.4%	37	8.2%	1.6	437.9	6.2%	295.6	8.6%	20–22	32–34	20–22	32–34	35–44
Jiangwai	29.9	3.5%	12	2.7%	2.5	179.6	2.5%	72.1	2.1%	20–23	32–34	20–23	32–34	46–69
Zhoushan	307.3	35.7%	179.5	40%	1.9	1843.9	26.1%	1076.7	31.2%	21–23	33–34	21–22	33–34	50–60
Zhouwai	62.2	7.2%	36.4	8.1%	2.2	435.7	6.2%	255.1	7.4%	21–23	33–34	21–23	33–35	57–84
Yushan	73.8	8.6%	47.5	10.6%	2.4	590.2	8.4%	380.3	11%	21–23	33–34	18–22	33–35	65–102
Wentai	7.4	0.9%	4	0.9%	2.1	29.7	0.4%	16	0.5%	22–25	32–35	18–23	34–35	47–115
	January, 2019 (Winter)													
Haizhou Bay	424.5	44.5%	82	26.7%	5.2	849	15.4%	164	8.2%	11–12	31–32	11–12	31–32	38
Lianqingshi	70.3	7.4%	35.4	11.6%	2.9	351.7	6.4%	177.2	8.9%	11–12	31–33	11–12	32–33	49–75
Lvsi	52	5.5%	11.4	3.7%	3.6	416.1	7.6%	91	4.6%	8–12	31–33	8–13	31–33	11–40
Dasha	172.7	18.1%	56.5	18.4%	3.6	2417.6	43.9%	791.4	39.7%	10–14	31–33	11–14	31–33	29–74
Yangtze River Estuary	43.6	4.6%	16.9	5.5%	2.3	174.4	3.2%	67.5	3.4%	11–13	32–34	12–14	32–34	35–44
Jiangwai	31	3.3%	15.4	5%	1.7	155.1	2.8%	77.2	3.9%	13–18	33–34	13–18	33–35	48–99

Zhoushan	76.2	8%	38.4	12.5%	2.2	686.1	12.5%	346	17.4%	14–17	33–35	14–17	33–35	41–66
Zhouwai	38.9	4.1%	24.2	7.9%	1.8	233.5	4.2%	145	7.3%	15–19	33–35	15–20	33–35	60–100
Yushan	44.7	4.7%	26.6	8.7%	1.7	223.3	4.1%	132.8	6.7%	15–19	34–35	15–19	34–35	46–114
May, 2019 (Spring)														
Haizhou Bay	116.3	4.1%	36	3%	3.4	232.5	1%	71.9	0.7%	14–15	30–32	14–15	30–32	20
Lianqingshi	210.9	7.5%	105.1	8.7%	2.3	1686.8	6.9%	841.1	8%	13–14	32–33	10–11	32–34	49–82
Lvsi	1985.7	70.7%	890.9	73.5%	2.3	17871.5	73.3%	8017.8	75.9%	13–16	30–33	12–16	30–33	13–39
Dasha	257.4	9.2%	89.1	7.4%	3.3	3346	13.7%	1158.8	11%	13–17	30–34	10–16	31–34	32–70
Yangtze River Estuary	172	6.1%	52.6	4.3%	3.7	860.2	3.5%	263.1	2.5%	13–18	30–33	13–18	30–33	22–43
Jiangwai	7.9	0.3%	4.1	0.3%	1.9	23.6	0.1%	12.3	0.1%	13–14	32.9	11–12	33–34	50–66
Zhoushan	11.1	0.4%	3	0.2%	2.5	120	0.5%	37.9	0.4%	15–20	29–34	14–20	31–34	26–76
Zhouwai	49.2	1.8%	31.2	2.6%	1.7	245.9	1%	155.8	1.5%	14–17	31–34	13–16	33–35	55–103
August, 2019 (Summer)														
Haizhou Bay	28.9	0.5%	4.7	0.6%	6.5	57.9	0.3%	9.5	0.3%	24–25	31–32	19–23	31–32	17–30
Dasha	140.7	2.6%	40	5.3%	3.9	985.1	4.8%	280.2	8.8%	26–28	29–31	18–26	29–32	23–45
Yangtze River Estuary	1593.5	29.8%	429.1	57.2%	4.2	4780.6	23.2%	1287.3	40.2%	27–28	23–32	22–24	32–33	22–30
Jiangwai	149.5	2.8%	28.5	3.8%	5.2	896.9	4.3%	171.3	5.3%	28–29	28–31	13–21	31–34	43–77
Zhoushan	478	8.9%	87.4	11.6%	5.3	3346.1	16.2%	612	19.1%	25–30	16–34	19–25	32–35	18–60
Zhouwai	448.5	8.4%	68.2	9.1%	6.2	2242.6	10.9%	340.9	10.6%	28–30	30–32	18–24	34–35	60–97
Yushan	96.2	1.8%	30.1	4%	5	673.7	3.3%	210.5	6.6%	26–29	29–34	20–28	31–35	31–71
Yuwai	2337.5	43.7%	45.5	6.1%	24.3	7012.4	34%	136.5	4.3%	28–29	33–34	18–20	35	86–104
Wentai–Mindong	69.9	1.3%	17.1	2.3%	4.3	629.2	3.1%	153.9	4.8%	25–29	30–34	18–28	32–35	43–101

					May, 2020 (Spring)									
Lianqingshi	132.6	4.4%	36.1	4.6%	4.5	928	5%	252.4	4.9%	12–21	31–34	10–20	31–34	15–70
Dasha	438.6	14.5%	155	19.9%	3.2	6140	32.9%	2169.5	42.3%	19–22	29–33	11–20	30–34	13–74
Yangtze River estuary	537.6	17.8%	94.6	12.2%	4.5	1612.8	8.7%	283.7	5.5%	20–23	26–32	17–21	31–32	10–40
Jiangwai	7.9	0.3%	8	1%	1.9	15.8	0.1%	16	0.3%	21–22	32–33	13–14	33–34	66–72
Zhoushan	296.8	9.8%	46.7	6%	6.1	2077.5	11.1%	326.8	6.4%	19–25	31–33	17–21	32–35	27–63
Zhouwai	489.9	16.2%	104.9	13.5%	5.5	2939.4	15.8%	629.5	12.3%	18–23	31–34	15–17	32–34	46–83
Yushan	428	14.1%	125.6	16.2%	3.3	2140.1	11.5%	628	12.2%	19–23	31–34	18–23	34–35	32–70
Yuwai	315.5	10.4%	97	12.5%	3.1	1262	6.8%	388	7.6%	19–23	32–34	17–21	33–35	30–90
Wentai	381.1	12.6%	109	14%	2.9	1524.4	8.2%	436	8.5%	23–25	32–35	20–23	34–35	46–50
					August, 2020 (Summer)									
Lianqingshi	36.3	0.6%	7.6	0.2%	4.7	72.7	0.2%	15.2	0.1%	24–25	30–32	10–25	30–33	15–72
Dasha	500.9	8%	365.4	12%	2.6	7012	17.3%	5115.9	24.6%	24–27	27–32	11–26	29–34	17–67
Yangtze River estuary	1327.4	21.2%	615.6	20.2%	2.2	11946.7	29.5%	5540.6	26.6%	24–25	22–33	22–25	31–33	18–45
Jiangwai	1172.1	18.8%	550	18.1%	2	5860.3	14.5%	2750.2	13.2%	24–25	30–33	18–25	32–35	28–80
Zhoushan	1931.5	30.9%	1147.1	37.7%	2.3	9657.3	23.8%	5735.5	27.5%	24–26	27–34	21–25	33–35	32–50
Zhouwai	316.1	5.1%	87.8	2.9%	4.5	1264.3	3.1%	351.1	1.7%	24–26	33–34	20–24	33–35	44–67
Yushan	724.3	11.6%	193.4	6.4%	4.6	3621.5	8.9%	966.8	4.6%	25–30	32–34	17–27	33–35	31–82
Yuwai	28.1	0.4%	19.5	0.6%	4.8	168.5	0.4%	117	0.6%	28–30	33–34	12–25	34–35	60–112
Wentai	130.1	2.1%	33.5	1.1%	5.4	650.3	1.6%	167.3	0.8%	28–30	33–35	20–26	34–35	30–100
Mindong	83.1	1.3%	24.3	0.8%	2.4	249.3	0.6%	73	0.4%	27–30	34–35	17–26	34–35	40–90
					May, 2021 (Spring)									
Lianqingshi	20.8	2%	4.3	2.4%	6.4	124.5	2.1%	25.7	2.3%	19–22	32–33	9–11	32–34	44–73
Dasha	473.1	45.2%	98.5	55.9%	5	4257.7	73.3%	886.2	78.4%	17–23	22–32	12–19	30–34	24–53

Yangtze River estuary	78.2	7.5%	5.3	3%	11.8	156.3	2.7%	10.5	0.9%	15–18	29–33	14–16	32–34	27–33
Zhoushan	107.6	10.3%	23.9	13.5%	6.3	538.1	9.3%	119.3	10.5%	18–20	29–32	17–19	33–35	27–50
Yushan	173.8	16.6%	24.7	14.0%	6	347.6	6%	49.5	4.4%	22–24	30–34	18–19	34–35	53–82
Wentai	192.9	18.4%	19.7	11.2%	9.4	385.8	6.6%	39.4	3.5%	23–25	33–34	17–18	34–35	75–82
November, 2021 (Autumn)														
Lvsi	141.2	3.7%	34.3	2.1%	6.2	706.2	3%	171.6	1.6%	8–12	30–32	8–12	30–32	14–40
Dasha	236.1	6.2%	163.4	10.2%	1.9	2597.4	10.9%	1797.1	16.8%	9–26	30–33	11–19	30–34	30–78
Yangtze River Estuary	44.5	1.2%	18.3	1.1%	2.7	311.6	1.3%	128.1	1.2%	12–17	31–33	13–17	31–33	29–43
Jiangwai	30.7	0.8%	16.5	1%	2.1	153.6	0.6%	82.4	0.8%	15–18	30–34	15–19	31–34	40–86
Zhoushan	388.9	10.2%	144.1	9%	2.9	1944.7	8.1%	720.7	6.7%	17–24	32–34	17–22	33–35	40–60
Zhouwai	734	19.2%	210.3	13.1%	1.7	3670	15.3%	1051.4	9.8%	17–24	32–34	17–22	32–35	49–90
Yushan	1954.4	51.2%	926.6	57.8%	2.7	13680.7	57.2%	6486.5	60.6%	20–24	30–34	19–24	33–35	50–80
Wentai	283.6	7.4%	89.8	5.6%	3.7	850.7	3.6%	269.3	2.5%	21–25	31–34	23–25	33–34	36–53
	Charybdis japonica													
November, 2018 (Autumn)														
Dasha	39	56.6%	4	18.2%	12.6	155.9	66%	15.8	25.2%	18–20	31–32	18–20	30–32	28–38
Yangtze River Estuary	20.4	29.7%	11.3	51.8%	1.7	61.3	26%	33.9	53.9%	20–22	32–33	20–22	32–34	35–44
Zhouwai	9.5	13.8%	6.6	30.2%	1.7	19	8%	13.2	20.9%	22–23	33–34	22–23	33–34	60–66
January, 2019 (Winter)														
Dasha	19.8	62.5%	3.4	30.7%	10.1	79.3	76.9%	13.8	46.8%	11–13	32–33	10–14	32–33	33–48
Yangtze River Estuary	11.9	37.5%	7.8	69.7%	1.5	23.8	23%	15.6	53.1%	13–14	33–34	13–14	33–34	35–39
May, 2019 (Spring)														
Dasha–Yangtze River Estuary	115.2	100%	7.6	100%	14.3	460.8	100%	30.6	100%	13–16	31–33	12–15	32–34	28–50
August, 2019 (Summer)														

Yangtze River Estuary	271.2	100%	12	100%	22.6	271.2	100%	12	100%	28	24	24	32	28
	May, 2020 (Spring)													
Yangtze River Estuary	207.8	100%	3.4	100%	60.6	207.8	100%	3.4	100%	22	31	21	32	27
	August, 2020 (Summer)													
Haizhou Bay	111	10.3%	1.5	4.4%	74	111	10.3%	1.5	4.4%	24	31	24	31	17
Yangtze River Estuary	44.5	4.1%	22.3	65%	2	44.5	4.1%	22.3	65%	24	26	25	30	24
Wentai	142.2	13.2%	3	8.8%	47.4	142.2	13.2%	3	8.8%	30	34	18	35	104
Mindong	778.8	72.3%	7.5	21.9%	90.1	778.8	72.3%	7.5	21.9%	29–30	33–34	16–28	34–35	70–100
	May, 2021 (Spring)													
Dasha	236.2	37.3%	10.9	39.8%	19.5	708.5	47.2%	32.8	49.8%	17–20	28–31	12–16	30–33	22–42
Yangtze River Estuary	396.3	62.7%	16.5	60.2%	19.5	792.5	52.8%	33	50.2%	16–19	29–33	13–16	31–33	32–33
	November, 2021 (Autumn)													
Dasha	53.1	38.2%	11.3	78.6%	47.9	106.2	38.2%	22.6	78.6%	12–13	31–32	12–13	31–33	30–64
Jiangwai	85.9	61.8%	3.1	21.4%	40.7	171.8	61.8%	6.2	21.4%	15–18	31–34	15–19	31–34	43–84
	Charybdis miles													
	November, 2018 (Autumn)													
Yushan	271.8	100%	2	100%	134	815.5	100%	6	100%	22–24	34–35	19–21	34–35	73–100
	January, 2019 (Winter)													
Yushan	282	100%	1.5	100%	198.9	564.1	100%	3	100%	16–17	34–35	16–18	34–35	78–92
	August, 2019 (Summer)													
Yushan	188.1	100%	3.8	100%	49.5	188.1	100%	3.8	100%	28.5	33.5	23.6	34.2	70
	May, 2020 (Spring)													
Zhouwai	629.3	68.1%	9.5	66.5%	98.6	3146.3	72.7%	47.7	71.3%	18–23	31–34	15–18	33–35	60–80
Yushan	294.7	31.9%	4.8	33.5%	92.8	1179	27.3%	19.2	28.7%	20–25	31–35	18–24	34–35	40–75

					August, 2020 (Summer)									
Haizhou Bay	87	4.5%	2	7%	43.5	87	1.1%	2	1.8%	26	31	26	31	8
Zhouwai	1077.4	55.1%	16.8	58.6%	68.7	4309.8	55.1%	67	60.3%	29–30	28–31	18–21	34–35	70–95
Yuwai	525.6	26.9%	7.1	24.7%	75	2102.3	26.9%	28.3	25.4%	28–30	33–34	20–25	34–35	64–80
Wentai	264	13.5%	2.8	9.7%	99.1	1320.2	16.9%	13.9	12.5%	29–30	34–35	16–26	34–35	60–110
					May, 2021 (Spring)									
Zhoushan	1130.1	62.4%	20	72.8%	77.3	5650.6	46.5%	99.9	58.3%	18–22	31–33	17–19	33–34	27–72
Yushan	382.2	21.1%	4.2	15.3%	107	3822.2	31.4%	41.9	24.5%	20–23	30–34	17–23	33–35	40–80
Wentai	298.1	16.5%	3.3	12%	103	2682.8	22.1%	29.5	17.2%	23–25	32–35	17–23	34–35	43–85
					November, 2021 (Autumn)									
Dasha	12.7	4.3%	0.9	23.5%	13.8	12.7	4.3%	0.9	23.5%	10	31	10	31	23
Zhoushan	212.3	72.2%	2	51%	106.2	212.3	72.2%	2	51%	23	33	20	34	65
Wentai	69	23.5%	1	25.5%	69	69	23.5%	1	25.5%	23	34	23	34	70

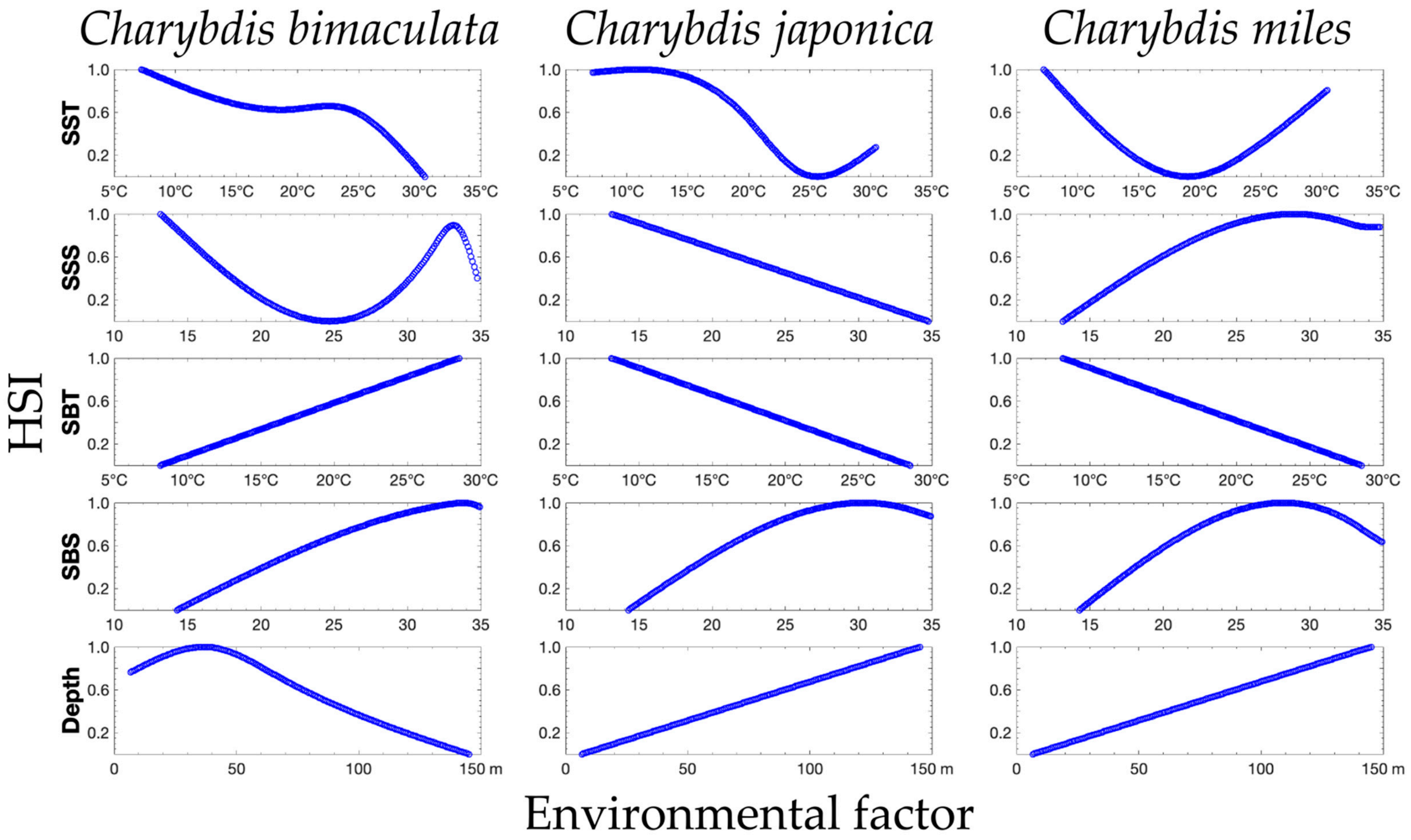

Figure S1. Habitat suitability response curves of three Charybdis species (*C. bimaculata*, *C. japonica*, and *C. miles*) against key environmental variables, including sea surface temperature (SST), sea surface salinity (SSS), sea bottom temperature (SBT), sea bottom salinity (SBS), and depth. The y-axis represents habitat suitability values ranging from 0 to 1, while the x-axis indicates the gradient of each corresponding environmental factor.

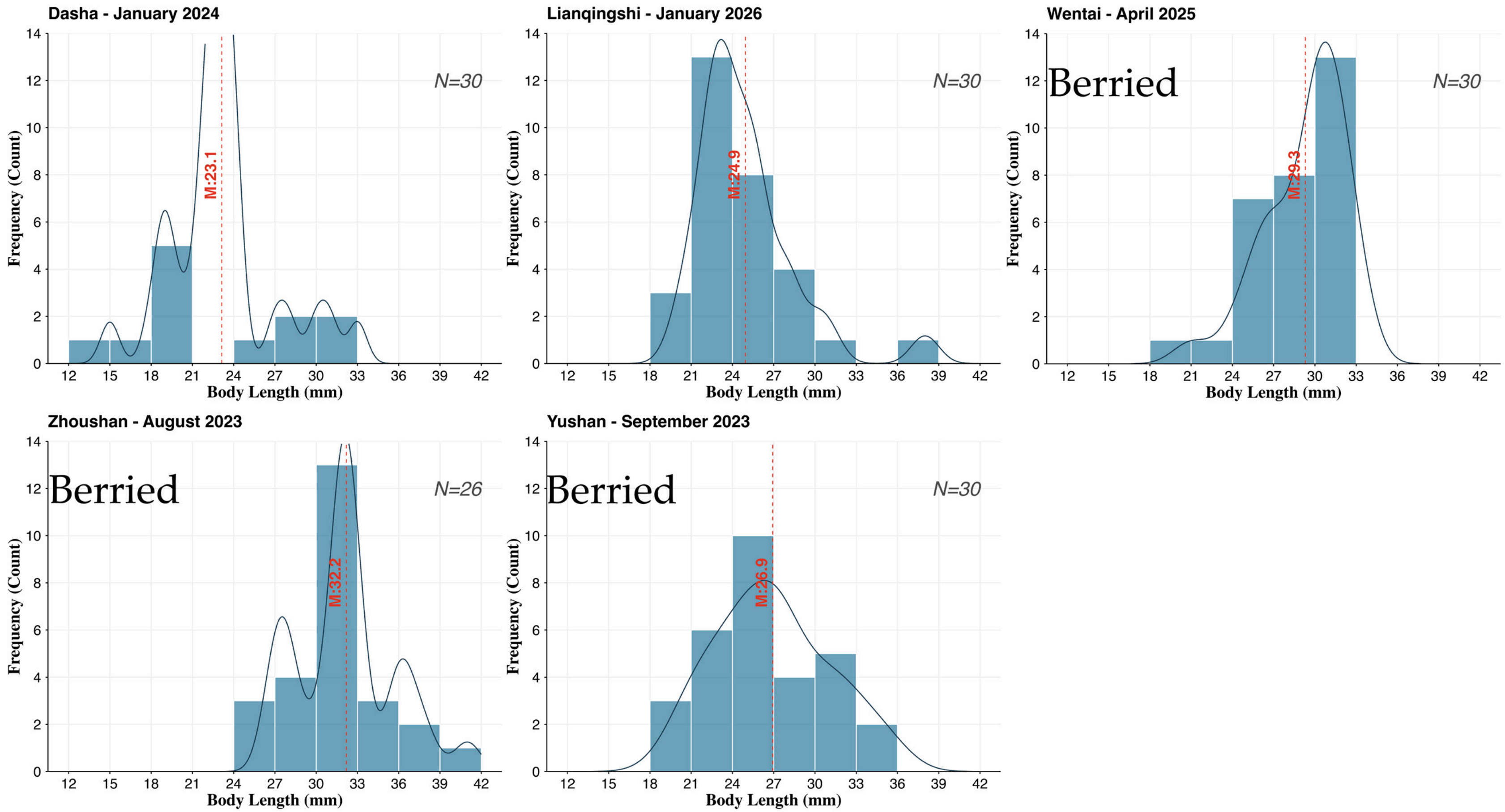

Figure S2 Length-frequency distributions of female crabs of *Charybdis bimaculata* collected at five sampling events covering multiple locations (Lianqingshi, Dasha, Zhoushan, Yushan, Wentai) from 2023 to 2026. Each panel presents the count of individuals across 12–42 mm body length intervals. Sampling location, month, year and sample size (N) are indicated above each histogram. The label "Berried" marks the presence of ovigerous females within the corresponding sample batch.

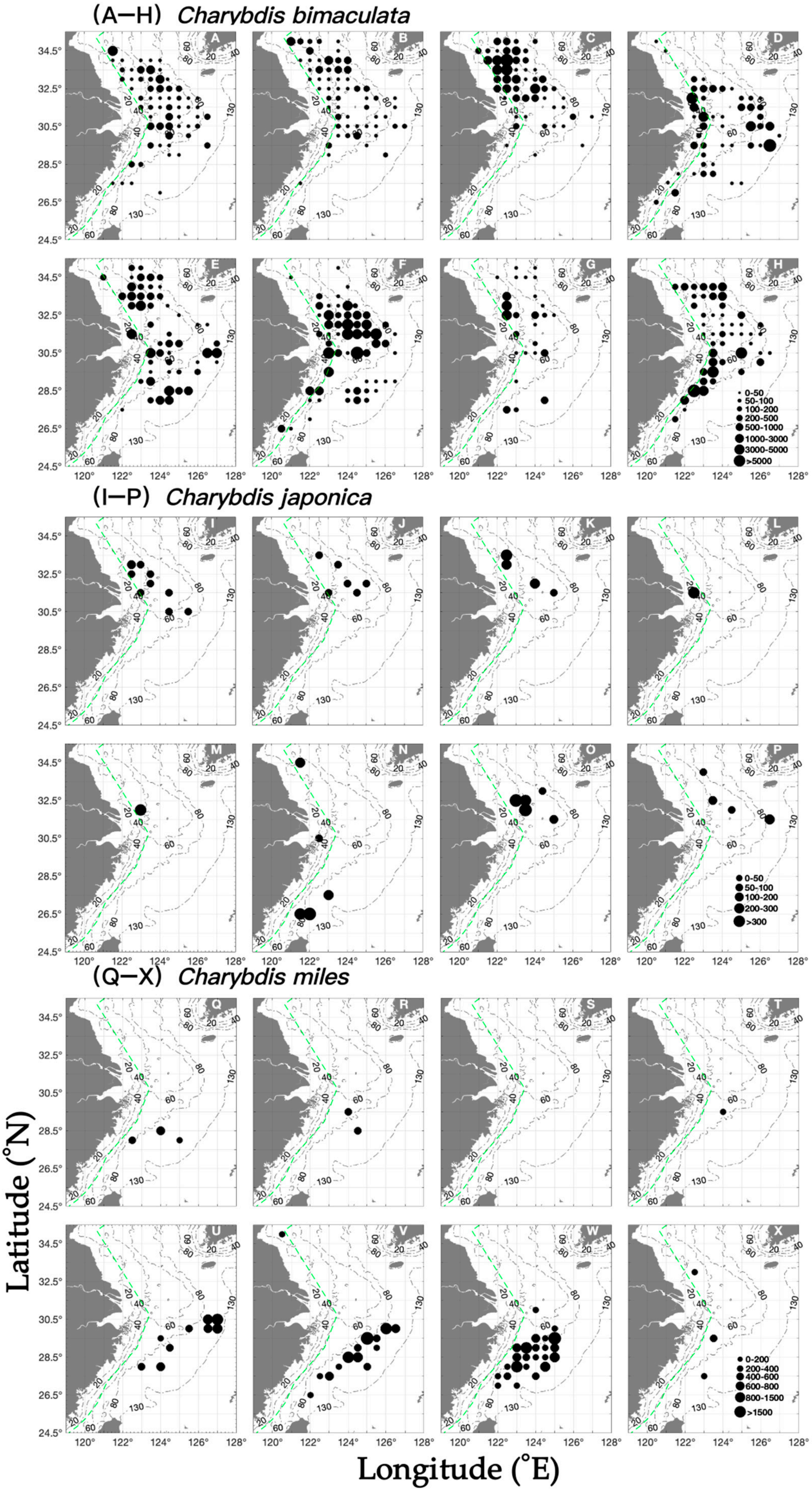

(A—H) Charybdis bimaculata
(I—P) Charybdis japonica
(Q—X) Charybdis miles
0-50
50-100
100-200
200-500
500-1000
1000-3000
3000-5000
>5000
0-50
50-100
100-200
200-300
>300
0-200
200-400
400-600
600-800
800-1500
>1500
Latitude (°N)
Longitude (°E)

Figure S3. Seasonal distribution patterns of $CPUE_w$ (unit: $g \cdot h^{-1}$) for three *Charybdis* crab species. (A–H) *Charybdis (Gonioneptunus) bimaculata* (Miers, 1886); $CPUE_w$ values were categorised into magnitude classes: 0–50, 50–100, 100–200, 200–500, 500–1000, 1000–3000, 3000–5000, and >5000 $g \cdot h^{-1}$. (I–P) *Charybdis (Charybdis) japonica* (A. Milne-Edwards, 1861); $CPUE_w$ magnitude classes: 0–50, 50–100, 100–200, 200–300, and >300 $g \cdot h^{-1}$. (Q–X) *Charybdis (Charybdis) miles* (De Haan, 1835); $CPUE_w$ magnitude classes: 0–200, 200–400, 400–600, 600–800, 800–1500, and >1500 $g \cdot h^{-1}$. Sampling periods: (A, I, Q) November 2018, (B, J, R) January 2019, (C, K, S) May 2019, (D, L, T) August 2019, (E, M, U) May 2020, (F, N, V) August 2020, (G, O, W) May 2021, (H, P, X) November 2021.

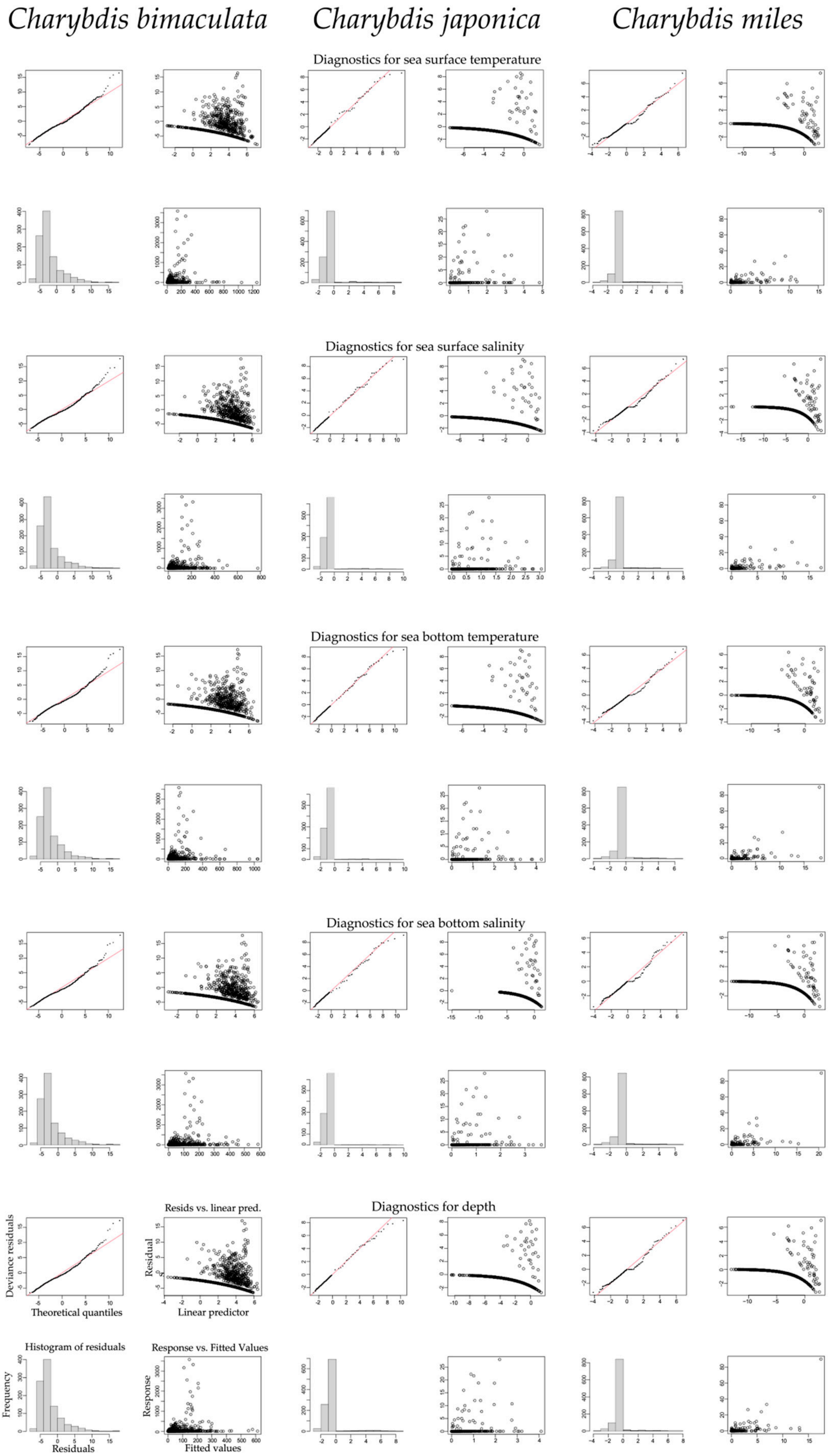
Charybdis bimaculata
Charybdis japonica
Charybdis miles
Diagnostics for sea surface temperature
Diagnostics for sea surface salinity
Diagnostics for sea bottom temperature
Diagnostics for sea bottom salinity
Diagnostics for depth
Deviance residuals
Theoretical quantiles
Resids vs. linear pred.
Residual
Linear predictor
Histogram of residuals
Frequency
Residuals
Response vs. Fitted Values
Response
Fitted values

Figure S4. Residual diagnostic plots for the generalized additive model of environmental variables such as sea surface temperature, sea surface salinity, sea bottom temperature, sea bottom salinity and depth for crab species *Charybdis bimaculata*, *Charybdis japonica*, and *Charybdis miles*. Each group contains four subgraphs: deviance residuals against linear predictors, Q-Q normal quantile plots, response versus fitted values, and histograms of residuals.

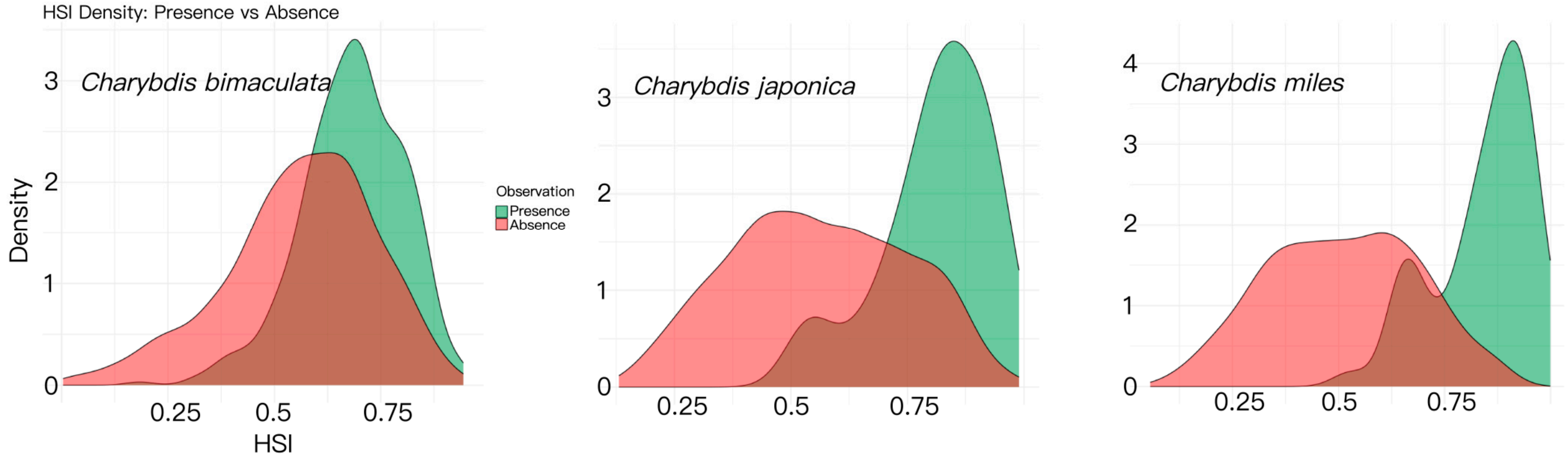

Figure S5. Habitat suitability index (HSI) density distributions for three Charybdis crab species: *Charybdis bimaculata*, *C. japonica*, and *C. miles*. Density curves are separated by observation status (presence vs. absence) to illustrate differences in HSI value frequency between sampling sites with and without target crab occurrence.
