# Supplementary material for: Spatiotemporal and Interannual Habitat Variability of Three Charybdis Swimming Crab Species (Brachyura: Portunidae) in the Southern Yellow Sea and East China Sea
Source: Biology (Basel). 2026 Aug 17;15(16):1406. doi: 10.3390/biology15161406 (PMC13509451; doi:10.3390/biology15161406)
Supplement: Supplementary file 1 [file biology-15-01406-s001.zip › Supplementary File S2 the code.pdf]

Supplementary File S2

```
options(stringsAsFactors = FALSE, warn = 1)
set.seed(123)

# ------------------------
# 0. 用户配置
# ------------------------
env_vars <- c("sst", "sss", "bt", "bs", "depth")
target_var <- "number"                    #  可改为  "biomass"
random_seed <- 123
train_fraction <- 0.80
main_brt_folds <- 10
cv_inner_brt_folds <- 5
spatial_cv_folds <- 5
bootstrap_replicates <- 50
refit_brt_weights_inside_cv <- TRUE    # TRUE  可避免  CV  权重信息泄漏
input_crs <- 4326                         # lon/lat  通常为  WGS84；若不是，请修改
install_missing_packages <- TRUE
install_optional_spatial_packages <- TRUE

get_desktop <- function() {
  if (.Platform$OS.type == "windows") {
    file.path(Sys.getenv("USERPROFILE"), "Desktop")
  } else {
    path.expand("~/Desktop")
  }
}

args <- commandArgs(trailingOnly = TRUE)
default_candidates <- c(
  file.path(get_desktop(), "wushixun.csv"),
  file.path(getwd(), "wushixun.csv")
)
input_file <- if (length(args) >= 1L) args[[1]] else {
  hit <- default_candidates[file.exists(default_candidates)]
  if (length(hit)) hit[[1]] else default_candidates[[1]]
}
output_dir <- if (length(args) >= 2L) args[[2]] else {
  file.path(get_desktop(), "GAM_BRT_HSI_output")
}
dir.create(output_dir, recursive = TRUE, showWarnings = FALSE)

out_file <- function(name) file.path(output_dir, name)
```

```
log_file <- out_file("model_run_log.txt")
cat("===== Model Running Log =====\n", file = log_file)

log_msg <- function(...) {
  txt <- paste0(..., collapse = "")
  cat(txt, "\n")
  cat(txt, "\n", file = log_file, append = TRUE)
}

log_msg("Run time: ", Sys.time())
log_msg("Random seed: ", random_seed)
log_msg("Input file: ", normalizePath(input_file, winslash = "/", mustWork = FALSE))
log_msg("Output directory: ", normalizePath(output_dir, winslash = "/", mustWork =
FALSE))

# -------------------------
# 1. 安装并加载包
# -------------------------
required_packages <- c("mgcv", "dismo", "gbm", "ggplot2", "dplyr", "pROC")
missing_required <- required_packages[!vapply(required_packages, requireNamespace,
logical(1), quietly = TRUE)]
if (length(missing_required) && install_missing_packages) {
  install.packages(
    missing_required,
    dependencies = c("Depends", "Imports", "LinkingTo"),
    repos = "https://cloud.r-project.org"
  )
}
missing_required <- required_packages[!vapply(required_packages, requireNamespace,
logical(1), quietly = TRUE)]
if (length(missing_required)) {
  stop("缺少必要 R 包：", paste(missing_required, collapse = ", "),
       "。请先运行 install.packages() 安装。")
}

invisible(lapply(required_packages, library, character.only = TRUE))

if (install_optional_spatial_packages) {
  optional_missing <- c("sf", "blockCV")[
    !vapply(c("sf", "blockCV"), requireNamespace, logical(1), quietly = TRUE)
  ]
  if (length(optional_missing) && install_missing_packages) {
    try(install.packages(
      optional_missing,
```

```r
      dependencies = c("Depends", "Imports", "LinkingTo"),
      repos = "https://cloud.r-project.org"
    ), silent = TRUE)
  }
}
has_blockcv <- requireNamespace("sf", quietly = TRUE) &&
  requireNamespace("blockCV", quietly = TRUE)
log_msg("blockCV available: ", has_blockcv)

# ------------------------
# 2. 通用辅助函数
# ------------------------
clamp01 <- function(x) pmin(1, pmax(0, x))

safe_mean <- function(x) {
  x <- x[is.finite(x)]
  if (!length(x)) NA_real_ else mean(x)
}

safe_sd <- function(x) {
  x <- x[is.finite(x)]
  if (length(x) < 2L) NA_real_ else stats::sd(x)
}

safe_cor <- function(x, y, method = "pearson") {
  ok <- is.finite(x) & is.finite(y)
  if (sum(ok) < 3L || length(unique(x[ok])) < 2L || length(unique(y[ok])) < 2L) {
    return(NA_real_)
  }
  suppressWarnings(stats::cor(x[ok], y[ok], method = method))
}

safe_auc <- function(labels, scores) {
  labels <- as.integer(labels)
  ok <- is.finite(labels) & is.finite(scores)
  if (sum(ok) < 3L || length(unique(labels[ok])) < 2L) return(NA_real_)
  as.numeric(pROC::auc(pROC::roc(labels[ok], scores[ok], quiet = TRUE,
                                 direction = "<")))
}

scale_with_bounds <- function(x, lower, upper, clamp = TRUE) {
  if (!is.finite(lower) || !is.finite(upper) || abs(upper - lower) < .Machine$double.eps^0.5)
{
    ans <- rep(0.5, length(x))
```

```
  } else {
    ans <- (x - lower) / (upper - lower)
  }
  if (clamp) ans <- clamp01(ans)
  ans
}

align_factor_levels <- function(newdata, traindata) {
  for (nm in intersect(names(newdata), names(traindata))) {
    if (is.factor(traindata[[nm]])) {
      newdata[[nm]] <- factor(as.character(newdata[[nm]]),
                              levels = levels(traindata[[nm]]))
    }
  }
  newdata
}

formula_to_text <- function(formula_object) {
  paste(deparse(formula_object, width.cutoff = 500L), collapse = "")
}

make_gam_formula <- function(var, data, include_year_re = TRUE,
                             include_season = TRUE, include_space = TRUE) {
  terms <- character(0)

  n_unique_var <- length(unique(data[[var]][is.finite(data[[var]])]))
  if (n_unique_var >= 4L) {
    k_env <- min(4L, n_unique_var - 1L)
    terms <- c(terms, sprintf("s(%s, k=%d, bs='cr')", var, k_env))
  } else {
    terms <- c(terms, var)
  }

  n_unique_xy <- nrow(unique(data[, c("lon", "lat"), drop = FALSE]))
  if (include_space && n_unique_xy >= 6L && nrow(data) >= 20L) {
    k_space <- min(20L, n_unique_xy - 1L, max(5L, floor(nrow(data) / 5L)))
    k_space <- max(4L, k_space)
    terms <- c(terms, sprintf("s(lon, lat, k=%d, bs='tp')", k_space))
  }

  if (include_season && length(unique(data$season)) >= 2L) {
    terms <- c(terms, "season")
  }
  if (include_year_re && length(unique(data$year)) >= 2L) {
```

```
    terms <- c(terms, "s(year, bs='re')")
  }

  stats::as.formula(paste("response ~", paste(terms, collapse = " + ")))
}

fit_si_gam <- function(data, var, include_year_re = TRUE,
                                   include_season = TRUE, include_space = TRUE) {
  form <- make_gam_formula(var, data, include_year_re,
                                       include_season, include_space)
  fit <- mgcv::gam(
    form,
    data = data,
    family = mgcv::tw(link = "log"),
    method = "REML",
    na.action = stats::na.exclude
  )
  train_link <- as.numeric(stats::predict(fit, newdata = data, type = "link"))
  list(
    model = fit,
    variable = var,
    formula = form,
    train_data = data,
    link_min = min(train_link, na.rm = TRUE),
    link_max = max(train_link, na.rm = TRUE)
  )
}

predict_si <- function(si_object, newdata, se.fit = FALSE) {
  nd <- align_factor_levels(newdata, si_object$train_data)
  if (se.fit) {
    pr <- stats::predict(si_object$model, newdata = nd,
                                    type = "link", se.fit = TRUE)
    return(list(
      fit = scale_with_bounds(as.numeric(pr$fit), si_object$link_min,
si_object$link_max),
      lower = scale_with_bounds(as.numeric(pr$fit - 1.96 * pr$se.fit),
                                          si_object$link_min, si_object$link_max),
      upper = scale_with_bounds(as.numeric(pr$fit + 1.96 * pr$se.fit),
                                          si_object$link_min, si_object$link_max)
    ))
  }
  lp <- as.numeric(stats::predict(si_object$model, newdata = nd, type = "link"))
  scale_with_bounds(lp, si_object$link_min, si_object$link_max)
```

```
}

get_gam_performance <- function(gam_mod) {
  obs <- as.numeric(gam_mod$model$response)
  fit <- as.numeric(stats::fitted(gam_mod))
  data.frame(
    deviance_explained = unname(summary(gam_mod)$dev.expl),
    AIC = AIC(gam_mod),
    BIC = BIC(gam_mod),
    RMSE = sqrt(mean((obs - fit)^2, na.rm = TRUE)),
    AUC_presence_absence = safe_auc(as.integer(obs > 0), fit),
    stringsAsFactors = FALSE
  )
}

get_concurvity_row <- function(gam_mod, var) {
  cm <- mgcv::concurvity(gam_mod, full = TRUE)
  term_candidates <- grep(paste0("^s\\(", var, "([,:\\)])"),
                          colnames(cm), value = TRUE)
  if (!length(term_candidates)) {
    return(data.frame(
      Variable = var,
      smooth_term = NA_character_,
      worst_concurvity = NA_real_,
      observed_concurvity = NA_real_,
      estimated_concurvity = NA_real_
    ))
  }
  term_name <- term_candidates[[1]]
  pull_value <- function(row_name) {
    if (row_name %in% rownames(cm)) as.numeric(cm[row_name, term_name]) else
NA_real_
  }
  data.frame(
    Variable = var,
    smooth_term = term_name,
    worst_concurvity = pull_value("worst"),
    observed_concurvity = pull_value("observed"),
    estimated_concurvity = pull_value("estimate")
  )
}

get_gam_dispersion <- function(gam_mod, var) {
  ratio <- stats::deviance(gam_mod) / stats::df.residual(gam_mod)
```

```
  data.frame(
    Variable = var,
    tweedie_scale = gam_mod$scale,
    deviance = stats::deviance(gam_mod),
    df_residual = stats::df.residual(gam_mod),
    deviance_df_ratio = ratio,
    descriptive_flag_gt_1_2 = is.finite(ratio) && ratio > 1.2
  )
}

is_nonnegative_integer_response <- function(x) {
  all(is.finite(x)) && all(x >= 0) && all(abs(x - round(x)) < 1e-8)
}

available_brt_predictors <- function(data, env_vars) {
  out <- env_vars
  if (length(unique(data$year)) >= 2L) out <- c(out, "year")
  if (length(unique(data$season)) >= 2L) out <- c(out, "season")
  out
}

fit_brt_model <- function(data, env_vars, requested_folds = 10L,
                          learning_rate_poisson = 0.005,
                          learning_rate_gaussian = 0.01,
                          max_trees = 5000L,
                          verbose = FALSE) {
  predictors <- available_brt_predictors(data, env_vars)
  n_folds <- min(as.integer(requested_folds), max(2L, floor(nrow(data) / 8L)))
  if (nrow(data) < 20L || n_folds < 2L) stop("BRT 样本量不足。")

  preferred_family <- if (is_nonnegative_integer_response(data$response)) "poisson" else
"gaussian"
  families_to_try <- unique(c(preferred_family, "gaussian"))
  errors <- character(0)

  for (family_name in families_to_try) {
    lr <- if (family_name == "poisson") learning_rate_poisson else learning_rate_gaussian
    fit_try <- tryCatch(
      dismo::gbm.step(
        data = data,
        gbm.x = predictors,
        gbm.y = "response",
        family = family_name,
        tree.complexity = 5,
```

```
          learning.rate = lr,
          bag.fraction = 0.6,
          n.folds = n_folds,
          prev.stratify = FALSE,
          n.trees = 50,
          step.size = 50,
          max.trees = max_trees,
          plot.main = FALSE,
          plot.folds = FALSE,
          verbose = verbose,
          silent = !verbose
        ),
        error = function(e) e
      )
      if (!inherits(fit_try, "error") && !is.null(fit_try)) {
        return(list(
          model = fit_try,
          family = family_name,
          predictors = predictors,
          n_folds = n_folds,
          learning_rate = lr
        ))
      }
      error_text <- if (inherits(fit_try, "error")) conditionMessage(fit_try) else "returned
NULL"
      errors <- c(errors, paste0(family_name, ": ", error_text))
    }
    stop("BRT 拟合失败：", paste(errors, collapse = " | "))
}

extract_brt_weights <- function(brt_result, env_vars) {
    if (is.null(brt_result) || is.null(brt_result$model)) {
      return(data.frame(var = env_vars, rel.inf = 1, W = 1 / length(env_vars)))
    }
    imp <- brt_result$model$contributions
    if (is.null(imp)) imp <- gbm::summary.gbm(brt_result$model, plotit = FALSE)
    imp <- as.data.frame(imp)
    names(imp)[1:2] <- c("var", "rel.inf")
    result <- merge(data.frame(var = env_vars), imp[, c("var", "rel.inf")],
                    by = "var", all.x = TRUE, sort = FALSE)
    result <- result[match(env_vars, result$var), , drop = FALSE]
    result$rel.inf[is.na(result$rel.inf)] <- 0
    total <- sum(result$rel.inf)
    result$W <- if (is.finite(total) && total > 0) {
```

```
    result$rel.inf / total
  } else {
    rep(1 / length(env_vars), length(env_vars))
  }
  result
}

get_brt_performance <- function(brt_result, observed) {
  mod <- brt_result$model
  pred <- as.numeric(mod$fitted)
  best_trees <- as.integer(mod$gbm.call$best.trees)
  data.frame(
    optimal_tree_number = best_trees,
    cv_deviance_mean = as.numeric(mod$cv.statistics$deviance.mean),
    cv_deviance_se = as.numeric(mod$cv.statistics$deviance.se),
    training_mean_residual_deviance = as.numeric(mod$self.statistics$mean.resid),
    RMSE = sqrt(mean((observed - pred)^2, na.rm = TRUE)),
    AUC_presence_absence = safe_auc(as.integer(observed > 0), pred)
  )
}

get_brt_cv_curve <- function(brt_result) {
  mod <- brt_result$model
  n <- min(length(mod$trees.fitted), length(mod$cv.values))
  if (!n) return(data.frame())
  data.frame(
    tree_number = mod$trees.fitted[seq_len(n)],
    cross_validation_deviance = mod$cv.values[seq_len(n)],
    cross_validation_se = if (length(mod$cv.loss.ses) >= n) {
      mod$cv.loss.ses[seq_len(n)]
    } else {
      rep(NA_real_, n)
    }
  )
}

get_brt_dispersion <- function(brt_result, observed) {
  if (brt_result$family != "poisson") {
    return(data.frame(
      family = brt_result$family,
      pearson_chisq = NA_real_,
      df_residual = NA_real_,
      pearson_dispersion_ratio = NA_real_,
      descriptive_flag_gt_1_2 = NA
```

```
    ))
  }
  fitted_response <- pmax(as.numeric(brt_result$model$fitted), 1e-12)
  pearson_chisq <- sum((observed - fitted_response)^2 / fitted_response, na.rm = TRUE)
  df_resid <- length(observed) - length(brt_result$predictors) - 1L
  ratio <- pearson_chisq / df_resid
  data.frame(
    family = brt_result$family,
    pearson_chisq = pearson_chisq,
    df_residual = df_resid,
    pearson_dispersion_ratio = ratio,
    descriptive_flag_gt_1_2 = is.finite(ratio) && ratio > 1.2
  )
}

brt_bootstrap_predictions <- function(brt_result, train_data, newdata,
                                      n_boot = 50L, seed = 123L) {
  set.seed(seed)
  predictors <- brt_result$predictors
  family_name <- brt_result$family
  best_trees <- as.integer(brt_result$model$gbm.call$best.trees)
  tree_depth <- as.integer(brt_result$model$gbm.call$tree.complexity)
  shrinkage <- as.numeric(brt_result$model$gbm.call$learning.rate)
  bag_fraction <- as.numeric(brt_result$model$gbm.call$bag.fraction)

  nd <- align_factor_levels(newdata, train_data)
  nd <- nd[, predictors, drop = FALSE]
  pred_matrix <- matrix(NA_real_, nrow = nrow(nd), ncol = n_boot)

  for (b in seq_len(n_boot)) {
    idx <- sample(seq_len(nrow(train_data)), replace = TRUE)
    boot_dat <- train_data[idx, c(predictors, "response"), drop = FALSE]
    fit_b <- tryCatch(
      gbm::gbm(
        formula = response ~ .,
        data = boot_dat,
        distribution = family_name,
        n.trees = best_trees,
        interaction.depth = tree_depth,
        shrinkage = shrinkage,
        bag.fraction = bag_fraction,
        train.fraction = 1,
        verbose = FALSE
      ),
```

```
        error = function(e) NULL
      )
      if (!is.null(fit_b)) {
        pred_matrix[, b] <- tryCatch(
          as.numeric(stats::predict(fit_b, newdata = nd,
                                    n.trees = best_trees, type = "response")),
          error = function(e) rep(NA_real_, nrow(nd))
        )
      }
    }

    row_summary <- function(fun) {
      apply(pred_matrix, 1L, function(z) {
        z <- z[is.finite(z)]
        if (!length(z)) NA_real_ else fun(z)
      })
    }
    data.frame(
      brt_boot_mean = row_summary(mean),
      brt_boot_sd = row_summary(stats::sd),
      brt_boot_lower = row_summary(function(z) stats::quantile(z, 0.025, names =
FALSE)),
      brt_boot_upper = row_summary(function(z) stats::quantile(z, 0.975, names =
FALSE)),
      successful_bootstrap_models = apply(pred_matrix, 1L, function(z) sum(is.finite(z)))
    )
}

hsi_metrics <- function(test_data, hsi, train_response_max) {
    normalized_response <- test_data$response / max(train_response_max, 1e-12)
    data.frame(
      n_test = nrow(test_data),
      AUC_presence_absence = safe_auc(as.integer(test_data$response > 0), hsi),
      normalized_RMSE = sqrt(mean((normalized_response - hsi)^2, na.rm = TRUE)),
      pearson_cor_HSI_response = safe_cor(hsi, test_data$response, "pearson"),
      spearman_cor_HSI_response = safe_cor(hsi, test_data$response, "spearman")
    )
}

fit_hsi_fold <- function(train_data, test_data, env_vars,
                         include_year_re, global_weights = NULL,
                         refit_weights = TRUE) {
    if (nrow(train_data) < 20L || nrow(test_data) < 2L) return(NULL)
```

```
include_season <- all(unique(as.character(test_data$season)) %in%
                      unique(as.character(train_data$season)))
if (include_year_re) {
  include_year_re <- all(unique(as.character(test_data$year)) %in%
                         unique(as.character(train_data$year))) &&
    length(unique(train_data$year)) >= 2L
}

model_list <- list()
si_matrix <- matrix(NA_real_, nrow = nrow(test_data), ncol = length(env_vars),
                    dimnames = list(NULL, env_vars))
for (j in seq_along(env_vars)) {
  var <- env_vars[[j]]
  obj <- tryCatch(
    fit_si_gam(train_data, var, include_year_re = include_year_re,
               include_season = include_season, include_space = TRUE),
    error = function(e) NULL
  )
  if (is.null(obj)) return(NULL)
  model_list[[var]] <- obj
  si_matrix[, j] <- predict_si(obj, test_data)
}

if (refit_weights) {
  fold_brt <- tryCatch(
    fit_brt_model(train_data, env_vars,
                  requested_folds = cv_inner_brt_folds,
                  learning_rate_poisson = 0.01,
                  learning_rate_gaussian = 0.02,
                  max_trees = 2500L,
                  verbose = FALSE),
    error = function(e) NULL
  )
  fold_weights <- extract_brt_weights(fold_brt, env_vars)
} else {
  fold_weights <- global_weights
}
if (is.null(fold_weights) || !nrow(fold_weights)) {
  fold_weights <- data.frame(var = env_vars, rel.inf = 1,
                             W = 1 / length(env_vars))
}
fold_weights <- fold_weights[match(env_vars, fold_weights$var), , drop = FALSE]
hsi <- as.numeric(si_matrix %*% fold_weights$W)
list(
```

```
    metrics = hsi_metrics(test_data, hsi, max(train_data$response, na.rm = TRUE)),
    hsi = hsi,
    weights = fold_weights
  )
}

make_spatial_folds <- function(data, k = 5L, seed = 123L, crs = 4326) {
  k <- min(k, length(unique(paste(data$lon, data$lat))),
           max(2L, floor(nrow(data) / 8L)))
  if (k < 2L) stop("无法生成至少 2 个空间折。")

  if (has_blockcv) {
    result <- tryCatch({
      sf_points <- sf::st_as_sf(data, coords = c("lon", "lat"),
                                crs = crs, remove = FALSE)
      block_object <- blockCV::cv_spatial(
        x = sf_points,
        column = NULL,
        rows_cols = c(5L, 5L),
        k = k,
        hexagon = FALSE,
        selection = "random",
        iteration = 50L,
        seed = seed,
        progress = FALSE,
        report = FALSE,
        plot = FALSE
      )
      list(ids = block_object$folds_ids, method = "blockCV::cv_spatial")
    }, error = function(e) NULL)
    if (!is.null(result) && length(result$ids) == nrow(data)) return(result)
  }

  set.seed(seed)
  xy <- scale(as.matrix(data[, c("lon", "lat")]))
  km <- stats::kmeans(xy, centers = k, nstart = 50)
  list(ids = km$cluster, method = "k-means spatial-cluster fallback")
}

# ------------------------
# 3. 读取和检查数据
# ------------------------
if (!file.exists(input_file)) {
  stop(
```

```r
      "没有找到输入数据：", input_file, "\n",
      "请把 wushixun.csv 放到桌面，或这样运行：\n",
      "Rscript \"GAM_BRT 加权 HSI_修正版.R\" \"完整/输入路径.csv\" \"完整/输出文件夹\""
    )
}

df <- utils::read.csv(input_file, check.names = FALSE, fileEncoding = "UTF-8-BOM")
if (!(target_var %in% names(df))) {
  stop("响应变量列不存在：", target_var, "。现有列为：", paste(names(df), collapse = ", "))
}
if (target_var != "response" && "response" %in% names(df)) {
  stop("数据同时含 target_var 和 response 列，无法安全重命名。")
}
names(df)[names(df) == target_var] <- "response"

required_columns <- c(env_vars, "response", "year", "season", "lon", "lat")
missing_columns <- setdiff(required_columns, names(df))
if (length(missing_columns)) {
  stop("数据缺少必要列：", paste(missing_columns, collapse = ", "))
}

numeric_columns <- c(env_vars, "response", "lon", "lat")
for (nm in numeric_columns) {
  original_na <- sum(is.na(df[[nm]]))
  df[[nm]] <- suppressWarnings(as.numeric(df[[nm]]))
  new_na <- sum(is.na(df[[nm]]))
  if (new_na > original_na) {
    log_msg("Warning: column ", nm, " produced ", new_na - original_na,
            " additional NA values during numeric conversion.")
  }
}

n_raw <- nrow(df)
df_clean <- df[stats::complete.cases(df[, required_columns]), , drop = FALSE]
df_clean$year <- factor(df_clean$year)
df_clean$season <- factor(df_clean$season)
df_clean$is_zero <- as.integer(df_clean$response == 0)

if (any(df_clean$response < 0)) stop("response 中存在负值，Tweedie/Poisson 模型不适用。")
if (nrow(df_clean) < 30L) stop("完整样本少于 30，当前 GAM+BRT+CV 配置无法稳定运行。")
if (length(unique(df_clean$response)) < 2L) stop("response 没有变异，无法建模。")
```

```
sample_summary <- data.frame(
  raw_samples = n_raw,
  cleaned_samples = nrow(df_clean),
  samples_removed_during_cleaning = n_raw - nrow(df_clean),
  positive_catch_stations = sum(df_clean$response > 0),
  zero_catch_stations = sum(df_clean$response == 0)
)
utils::write.csv(sample_summary, out_file("sample_cleaning_statistics.csv"), row.names =
FALSE)

if ("species" %in% names(df_clean)) {
  species_obs_stats <- df_clean |>
    dplyr::group_by(species) |>
    dplyr::summarise(
      total_obs = dplyr::n(),
      positive_stations = sum(response > 0),
      zero_stations = sum(response == 0),
      .groups = "drop"
    )
} else {
  species_obs_stats <- data.frame(
    note = "No species column in dataset; global statistics only",
    total_obs_global = nrow(df_clean),
    positive_global = sum(df_clean$response > 0),
    zero_global = sum(df_clean$response == 0)
  )
}
utils::write.csv(species_obs_stats,                out_file("per_species_observation_stats.csv"),
row.names = FALSE)
log_msg("Clean samples: ", nrow(df_clean), "; removed: ", n_raw - nrow(df_clean))

# 分层留出：尽量保证每个 year × presence/absence 层在训练集中有记录
set.seed(random_seed)
strata <- interaction(df_clean$year, as.integer(df_clean$response > 0), drop = TRUE)
train_idx <- unlist(lapply(split(seq_len(nrow(df_clean)), strata), function(idx) {
  if (length(idx) <= 1L) return(idx)
  n_train_stratum <- min(length(idx) - 1L, max(1L, floor(train_fraction * length(idx))))
  sample(idx, n_train_stratum, replace = FALSE)
}), use.names = FALSE)
train_idx <- sort(unique(train_idx))
test_idx <- setdiff(seq_len(nrow(df_clean)), train_idx)

if (length(test_idx) < 5L) {
```

```r
  set.seed(random_seed)
  n_train <- min(nrow(df_clean) - 5L, floor(train_fraction * nrow(df_clean)))
  train_idx <- sort(sample(seq_len(nrow(df_clean)), n_train, replace = FALSE))
  test_idx <- setdiff(seq_len(nrow(df_clean)), train_idx)
}

df_train <- droplevels(df_clean[train_idx, , drop = FALSE])
df_holdout <- df_clean[test_idx, , drop = FALSE]
df_holdout$year <- factor(df_holdout$year, levels = levels(df_train$year))
df_holdout$season <- factor(df_holdout$season, levels = levels(df_train$season))

utils::write.csv(df_train, out_file("training_dataset_80pct.csv"), row.names = FALSE)
utils::write.csv(df_holdout,  out_file("holdout_validation_dataset_20pct.csv"),  row.names
= FALSE)
log_msg("Training n = ", nrow(df_train), "; holdout n = ", nrow(df_holdout))

# -------------------------
# 4. GAM 拟合、SI、诊断
# -------------------------
gam_models <- list()
concurvity_all <- data.frame()
gam_dispersion_all <- data.frame()
gam_metadata_all <- data.frame()
si_curve_list <- list()

 grDevices::pdf(out_file("GAM_Diagnostics_Concurvity_Dispersion.pdf"),
                width = 11, height = 8.5)
for (var in env_vars) {
  log_msg("Fitting GAM for ", var, " ...")
  si_obj <- fit_si_gam(df_train, var, include_year_re = TRUE,
                       include_season = TRUE, include_space = TRUE)
  gam_models[[var]] <- si_obj
  df_train[[paste0("SI_", var)]] <- predict_si(si_obj, df_train)

  conc_row <- get_concurvity_row(si_obj$model, var)
  concurvity_all <- dplyr::bind_rows(concurvity_all, conc_row)

  disp_row <- get_gam_dispersion(si_obj$model, var)
  gam_dispersion_all <- dplyr::bind_rows(gam_dispersion_all, disp_row)

  perf <- get_gam_performance(si_obj$model)
  meta <- data.frame(
    target_environment_var = var,
    full_model_formula = formula_to_text(si_obj$formula),
```

```
    response_distribution = "Tweedie",
    link_function = "log",
    perf,
    stringsAsFactors = FALSE
  )
  gam_metadata_all <- dplyr::bind_rows(gam_metadata_all, meta)

  try(mgcv::gam.check(si_obj$model), silent = TRUE)
  graphics::plot(stats::fitted(si_obj$model),
                 stats::residuals(si_obj$model, type = "pearson"),
                 pch = 16, cex = 0.45,
                 xlab = "Fitted response", ylab = "Pearson residual",
                 main = paste0("GAM residuals: ", var,
                               "\nDeviance/df = ",
round(disp_row$deviance_df_ratio, 3)))
  graphics::abline(h = c(-2, 0, 2), lty = c(2, 1, 2))

  eval_range <- seq(min(df_train[[var]], na.rm = TRUE),
                    max(df_train[[var]], na.rm = TRUE), length.out = 200L)
  pred_df <- data.frame(
    season = factor(levels(df_train$season)[1], levels = levels(df_train$season)),
    year = factor(levels(df_train$year)[1], levels = levels(df_train$year)),
    lon = mean(df_train$lon, na.rm = TRUE),
    lat = mean(df_train$lat, na.rm = TRUE)
  )
  pred_df <- pred_df[rep(1L, length(eval_range)), , drop = FALSE]
  pred_df[[var]] <- eval_range
  curve_pred <- predict_si(si_obj, pred_df, se.fit = TRUE)
  si_curve_list[[var]] <- data.frame(
    Variable = var,
    EnvValue = eval_range,
    SI = curve_pred$fit,
    Lower = curve_pred$lower,
    Upper = curve_pred$upper
  )
}
grDevices::dev.off()

si_data_long <- dplyr::bind_rows(si_curve_list)
utils::write.csv(concurvity_all, out_file("GAM_concurvity_assessment.csv"), row.names =
FALSE)
utils::write.csv(gam_dispersion_all, out_file("GAM_dispersion_assessment.csv"),
row.names = FALSE)
utils::write.csv(gam_metadata_all, out_file("GAM_full_model_metadata.csv"),
```

```r
row.names = FALSE)
utils::write.csv(si_data_long, out_file("SI_response_curve_data.csv"), row.names = FALSE)

# ------------------------
# 5. BRT 拟合与权重
# ------------------------
log_msg("Fitting main BRT ...")
brt_result <- tryCatch(
  fit_brt_model(df_train, env_vars,
                requested_folds = main_brt_folds,
                learning_rate_poisson = 0.005,
                learning_rate_gaussian = 0.01,
                max_trees = 5000L,
                verbose = FALSE),
  error = function(e) {
    log_msg("BRT failed; equal weights will be used. Error: ", conditionMessage(e))
    NULL
  }
)

weights <- extract_brt_weights(brt_result, env_vars)
utils::write.csv(weights, out_file("variable_importance_weights.csv"), row.names = FALSE)
log_msg("HSI weights: ", paste(weights$var, round(weights$W, 4), sep = "=", collapse = "; "))

if (!is.null(brt_result)) {
  brt_perf <- get_brt_performance(brt_result, df_train$response)
  brt_curve <- get_brt_cv_curve(brt_result)
  brt_disp <- get_brt_dispersion(brt_result, df_train$response)
  brt_meta <- data.frame(
    brt_predictors = paste(brt_result$predictors, collapse = ","),
    response_distribution = brt_result$family,
    cross_validation_folds = brt_result$n_folds,
    tree_complexity = brt_result$model$gbm.call$tree.complexity,
    learning_rate = brt_result$learning_rate,
    bag_fraction = brt_result$model$gbm.call$bag.fraction,
    optimal_number_of_trees = brt_result$model$gbm.call$best.trees
  )
  utils::write.csv(brt_perf, out_file("BRT_model_performance_metrics.csv"), row.names = FALSE)
  utils::write.csv(brt_curve, out_file("BRT_cross_validation_error_curve.csv"), row.names = FALSE)
```

```
  utils::write.csv(brt_disp,   out_file("BRT_dispersion_assessment.csv"),   row.names   =
FALSE)
  utils::write.csv(brt_meta,   out_file("BRT_full_model_metadata.csv"),   row.names   =
FALSE)

  grDevices::pdf(out_file("BRT_diagnostic_plots.pdf"), width = 9, height = 7)
  if (nrow(brt_curve)) {
    graphics::plot(brt_curve$tree_number, brt_curve$cross_validation_deviance,
                   type = "l", lwd = 2,
                   xlab = "Number of trees", ylab = "Cross-validation deviance",
                   main = "BRT cross-validation curve")
    graphics::abline(v = brt_result$model$gbm.call$best.trees, lty = 2)
  }
  brt_fitted <- as.numeric(brt_result$model$fitted)
  graphics::plot(brt_fitted, df_train$response - brt_fitted,
                 pch = 16, cex = 0.45,
                 xlab = "Fitted response", ylab = "Raw residual",
                 main = "BRT residuals vs fitted")
  graphics::abline(h = 0, lty = 2)
  grDevices::dev.off()
}

# ------------------------
# 6. 训练集与留出集 HSI
# ------------------------
weights <- weights[match(env_vars, weights$var), , drop = FALSE]
si_cols <- paste0("SI_", env_vars)
df_train$HSI <- as.numeric(as.matrix(df_train[, si_cols, drop = FALSE]) %*% weights$W)
df_train$Suitability <- cut(
  df_train$HSI,
  breaks = c(-Inf, 0.4, 0.7, Inf),
  labels = c("Suboptimal", "Moderate", "Optimal"),
  right = FALSE
)

holdout_si <- matrix(NA_real_, nrow = nrow(df_holdout), ncol = length(env_vars),
                     dimnames = list(NULL, si_cols))
for (j in seq_along(env_vars)) {
  var <- env_vars[[j]]
  holdout_si[, j] <- predict_si(gam_models[[var]], df_holdout)
  df_holdout[[paste0("SI_", var)]] <- holdout_si[, j]
}
df_holdout$HSI <- as.numeric(holdout_si %*% weights$W)
df_holdout$Suitability <- cut(
```

```
  df_holdout$HSI,
  breaks = c(-Inf, 0.4, 0.7, Inf),
  labels = c("Suboptimal", "Moderate", "Optimal"),
  right = FALSE
)

holdout_metrics <- hsi_metrics(df_holdout, df_holdout$HSI,
                               max(df_train$response, na.rm = TRUE))
names(holdout_metrics) <- paste0("holdout_", names(holdout_metrics))

if (!is.null(brt_result) && nrow(df_holdout) > 0L) {
  log_msg("Running BRT bootstrap prediction uncertainty (", bootstrap_replicates, " replicates) ...")
  boot_uncertainty <- brt_bootstrap_predictions(
    brt_result, df_train, df_holdout,
    n_boot = bootstrap_replicates, seed = random_seed
  )
  boot_output <- cbind(
    df_holdout[, intersect(c("year", "season", "lon", "lat", "response"), names(df_holdout)), drop = FALSE],
    boot_uncertainty
  )
  utils::write.csv(boot_output,
                   out_file("BRT_bootstrap_holdout_prediction_uncertainty.csv"),
                   row.names = FALSE)
}

# ------------------------
# 7. 时间交叉验证（留一年）
# ------------------------
temporal_fold_rows <- list()
years <- levels(droplevels(df_train$year))
if (length(years) >= 2L) {
  for (y in years) {
    temp_train <- droplevels(df_train[as.character(df_train$year) != y, , drop = FALSE])
    temp_test <- df_train[as.character(df_train$year) == y, , drop = FALSE]
    result <- tryCatch(
      fit_hsi_fold(
        temp_train, temp_test, env_vars,
        include_year_re = FALSE,
        global_weights = weights,
        refit_weights = refit_brt_weights_inside_cv
      ),
      error = function(e) NULL
```

```
      )
      if (!is.null(result)) {
        row <- cbind(held_out_year = y, result$metrics)
      } else {
        row <- data.frame(
          held_out_year = y, n_test = nrow(temp_test),
          AUC_presence_absence = NA_real_, normalized_RMSE = NA_real_,
          pearson_cor_HSI_response = NA_real_, spearman_cor_HSI_response =
NA_real_
        )
      }
      temporal_fold_rows[[y]] <- row
    }
}
temporal_fold_metrics <- if (length(temporal_fold_rows)) {
  dplyr::bind_rows(temporal_fold_rows)
} else {
  data.frame(
    held_out_year = NA_character_, n_test = NA_integer_,
    AUC_presence_absence = NA_real_, normalized_RMSE = NA_real_,
    pearson_cor_HSI_response = NA_real_, spearman_cor_HSI_response = NA_real_
  )
}
utils::write.csv(temporal_fold_metrics, out_file("HSI_temporal_CV_fold_metrics.csv"),
row.names = FALSE)

temporal_summary <- data.frame(
  temporal_cv_mean_AUC =
safe_mean(temporal_fold_metrics$AUC_presence_absence),
  temporal_cv_sd_AUC = safe_sd(temporal_fold_metrics$AUC_presence_absence),
  temporal_cv_mean_normalized_RMSE =
safe_mean(temporal_fold_metrics$normalized_RMSE),
  temporal_cv_sd_normalized_RMSE =
safe_sd(temporal_fold_metrics$normalized_RMSE),
  temporal_cv_mean_pearson =
safe_mean(temporal_fold_metrics$pearson_cor_HSI_response),
  temporal_cv_mean_spearman =
safe_mean(temporal_fold_metrics$spearman_cor_HSI_response)
)

# -------------------------
# 8. 空间交叉验证
# -------------------------
spatial_fold_rows <- list()
```

```
spatial_method <- NA_character_
spatial_folds <- tryCatch(
  make_spatial_folds(df_train, k = spatial_cv_folds,
                     seed = random_seed, crs = input_crs),
  error = function(e) NULL
)
if (!is.null(spatial_folds)) {
  spatial_method <- spatial_folds$method
  fold_ids <- spatial_folds$ids
  for (fold in sort(unique(fold_ids))) {
    sp_train <- droplevels(df_train[fold_ids != fold, , drop = FALSE])
    sp_test <- df_train[fold_ids == fold, , drop = FALSE]
    result <- tryCatch(
      fit_hsi_fold(
        sp_train, sp_test, env_vars,
        include_year_re = TRUE,
        global_weights = weights,
        refit_weights = refit_brt_weights_inside_cv
      ),
      error = function(e) NULL
    )
    if (!is.null(result)) {
      row <- cbind(spatial_fold = fold, result$metrics)
    } else {
      row <- data.frame(
        spatial_fold = fold, n_test = nrow(sp_test),
        AUC_presence_absence = NA_real_, normalized_RMSE = NA_real_,
        pearson_cor_HSI_response = NA_real_, spearman_cor_HSI_response =
NA_real_
      )
    }
    spatial_fold_rows[[as.character(fold)]] <- row
  }
}
spatial_fold_metrics <- if (length(spatial_fold_rows)) {
  dplyr::bind_rows(spatial_fold_rows)
} else {
  data.frame(
    spatial_fold = NA_integer_, n_test = NA_integer_,
    AUC_presence_absence = NA_real_, normalized_RMSE = NA_real_,
    pearson_cor_HSI_response = NA_real_, spearman_cor_HSI_response = NA_real_
  )
}
spatial_fold_metrics$fold_generation_method <- spatial_method
```

```r
utils::write.csv(spatial_fold_metrics, out_file("HSI_spatial_CV_fold_metrics.csv"), row.names = FALSE)

spatial_summary <- data.frame(
  spatial_cv_method = spatial_method,
  spatial_cv_mean_AUC = safe_mean(spatial_fold_metrics$AUC_presence_absence),
  spatial_cv_sd_AUC = safe_sd(spatial_fold_metrics$AUC_presence_absence),
  spatial_cv_mean_normalized_RMSE = safe_mean(spatial_fold_metrics$normalized_RMSE),
  spatial_cv_sd_normalized_RMSE = safe_sd(spatial_fold_metrics$normalized_RMSE),
  spatial_cv_mean_pearson = safe_mean(spatial_fold_metrics$pearson_cor_HSI_response),
  spatial_cv_mean_spearman = safe_mean(spatial_fold_metrics$spearman_cor_HSI_response),
  stringsAsFactors = FALSE
)

full_validation_summary <- cbind(holdout_metrics, temporal_summary, spatial_summary)
utils::write.csv(full_validation_summary,
                 out_file("HSI_full_robust_validation_metrics.csv"),
                 row.names = FALSE)

# ------------------------
# 9. 绘图与最终导出
# ------------------------
p1 <- ggplot2::ggplot(si_data_long, ggplot2::aes(x = EnvValue, y = SI)) +
  ggplot2::geom_ribbon(ggplot2::aes(ymin = Lower, ymax = Upper), alpha = 0.25) +
  ggplot2::geom_line(linewidth = 0.8) +
  ggplot2::facet_wrap(~Variable, scales = "free_x") +
  ggplot2::theme_bw() +
  ggplot2::labs(
    title = "SI response curves (GAM-Tweedie)",
    x = "Environmental value", y = "Suitability index"
  )

df_holdout$Observation <- ifelse(df_holdout$response > 0, "Presence", "Absence")
p2 <- ggplot2::ggplot(df_holdout, ggplot2::aes(x = HSI, fill = Observation)) +
  ggplot2::geom_histogram(position = "identity", alpha = 0.5, bins = 20) +
  ggplot2::theme_minimal() +
  ggplot2::labs(
    title = "HSI distribution in holdout data",
    x = "HSI", y = "Count", fill = "Observation"
  )
```

```
ggplot2::ggsave(out_file("SI_response_curves.pdf"), p1,
                width = 10, height = 8)
ggplot2::ggsave(out_file("HSI_holdout_validation_distribution.pdf"), p2,
                width = 8, height = 6)

utils::write.csv(df_train, out_file("training_data_with_SI_HSI.csv"), row.names = FALSE)
utils::write.csv(df_holdout, out_file("holdout_data_with_SI_HSI.csv"), row.names =
FALSE)

capture.output(sessionInfo(), file = out_file("R_sessionInfo.txt"))

manifest <- c(
  paste0("Input: ", normalizePath(input_file, winslash = "/", mustWork = FALSE)),
  paste0("Output: ", normalizePath(output_dir, winslash = "/", mustWork = FALSE)),
  paste0("Rows raw/clean/train/holdout: ", n_raw, "/", nrow(df_clean), "/",
         nrow(df_train), "/", nrow(df_holdout)),
  paste0("BRT family: ", if (is.null(brt_result)) "not fitted; equal weights" else
brt_result$family),
  paste0("Spatial CV: ", spatial_method),
  paste0("Completed: ", Sys.time())
)
writeLines(manifest, out_file("run_manifest.txt"))

log_msg("Full validation summary:")
log_msg(paste(capture.output(print(full_validation_summary)), collapse = "\n"))
log_msg("All analyses completed successfully.")
cat("\n运行完成。输出目录：", normalizePath(output_dir, winslash = "/", mustWork =
FALSE), "\n")
```
